# Structural Optimization of Pterostilbene, a Promising Lead Molecule, and Evaluation of Its Derivatives via ADMET Prediction and In Vitro/In Vivo Anti-Cerebral Ischemic Activity

**DOI:** 10.3390/ijms27104512

**Published:** 2026-05-18

**Authors:** Kecan Zhang, Jiaxin Li, Yanan Dai, Zhihong Yang

**Affiliations:** 1 Institute of Medicinal Plant Development, Chinese Academy of Medical Sciences, Peking Union Medical College, Beijing 100193, China; zhangkecan11@163.com (K.Z.); s2025009019@student.pumc.edu.cn (J.L.); daiyanan0604@outlook.com (Y.D.); 2 National Engineering Laboratory for Breeding of Endangered Medicinal Materials, Beijing 100193, China

**Keywords:** pterostilbene, derivatives, cerebral ischemia-reperfusion injury, cerebral endothelial protection, pharmacological evaluation, ADMET prediction, acute toxicity

## Abstract

Pterostilbene (Pts), a small molecule stilbenoid and a dimethyl analogue of the star molecule resveratrol, exerts significant blood–brain barrier protection on cerebral ischemia-reperfusion injury and has received extensive attention. This study performed structural optimizations on Pts to obtain a series of derivatives and investigated their anti-ischemic activities both in vitro and in vivo, aiming to identify candidates with high safety and improved efficacy compared with Pts. The ADMET method was used to predict the drug-likeness of a series of Pts derivatives, and in vitro MTT cell viability analysis was conducted on neuroblastoma cells (SH-SY5Y) and brain microvascular endothelial cells (BMECs) after oxygen-glucose deprivation/reperfusion (OGD/R) injury. On the basis of the cytotoxicity results, four derivatives (NO. **1**, NO. **3**, NO. **5**, and NO. **7**) were selected for subsequent in vitro and in vivo biological activities evaluation. These compounds exhibited significantly higher TI values (18.29–30.61) in OGD/R-injured hBMECs compared with Pts (7.63) and effectively suppressed apoptosis, promoted cell migration, and enhanced tube formation capacity. In vivo, NO. **3** (5 mg/kg, ip., 7 d) demonstrated superior efficacy compared to Pts in improving cerebral blood flow, reducing infarction volume, enhancing neurological function, and modulating serum biomarker levels in middle cerebral artery occlusion/reperfusion (MCAO/R) rats, whereas NO. **1** and NO. **7** showed comparable efficacy to Pts. The acute intraperitoneal toxicity of NO. **3** was conducted and showed that the LD_50_ of NO. **3** was estimated to be more than 300 mg/kg. In this study, the rational design and comprehensive evaluation of Pts derivatives were reported. Compound NO. **3** demonstrated superior pharmacological efficacy to Pts both in vitro and in vivo, and it may be a promising therapeutic candidate for ischemic stroke intervention.

## 1. Introduction

Stroke is the second leading cause of death worldwide, with approximately 13.7 million new cases and 5.5 million deaths each year. Ischemic stroke accounts for 87% of all types of strokes [1]. The key treatment goal for acute ischemic stroke is to restore cerebral blood flow (CBF) and reperfusion [2]. Current therapeutic strategies, including intravenous thrombolysis and mechanical thrombectomy, inevitably trigger cerebral ischemia-reperfusion injury (CIRI), thereby limiting the therapeutic effect [3]. Prior investigations have focused extensively on neuronal injury as a therapeutic target. In contrast, the positive results obtained from related interventions in animal models were difficult to be verified in clinical studies, resulting in limited efficacy of similar neuroprotective strategies in clinical trials [4]. In recent years, more and more studies have recognized that CIRI is not a single neuronal injury, but rather a complex process involving the coordinated imbalance of multiple components, such as endothelial cells and pericytes [5]. Brain microvascular endothelial cells (BMECs) are one of the main components of the neurovascular unit (NVU) and are a core structural foundation of the blood–brain barrier (BBB). BMECs suffer from multiple damages, such as oxidative stress, inflammatory infiltration, and excessive activation of the matrix metalloproteinases in the CIRI process, resulting in decreased migration ability, abnormal angiogenesis, and BBB repair ability [6]. BMECs occur substantial pathological alterations at the early phase of CIRI, indicating that BMECs may be a potential strategy for CIRI prevention and treatment [7].

**Figure 1:**
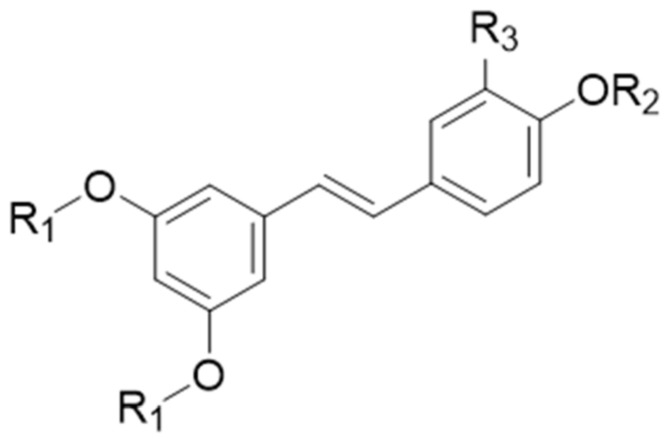
The core scaffold of pterostilbene (Pts) and its derivatives.

Natural products continue to serve as an invaluable reservoir for identifying and optimizing new therapeutic agents. Pterostilbene (Pts) is a small molecule stilbenoid that was first isolated from the heartwood of *Pterocarpus santalinus* and was soon discovered in grapes and blueberries [8,9]. Multiple studies have shown that Pts manifests significant protective effects in various pathological models, such as metabolic diseases [10], cardiovascular diseases [11], neurodegenerative diseases [12], and tumors [13], and that it possesses multiple pharmacological activities, including anti-inflammatory [14], antioxidant, and anti-apoptotic [15] activity, as well as BMEC protection [16]. Notably, Pts is also one of the principal stilbenoid constituents of Resina Draconis, a traditional medicinal material that has been officially approved as a pharmaceutical raw material in China. Resina Draconis is the primary component of the marketed formulations, including Longxuetongluo Capsule [17]. Tissue distribution studies revealed that Pts exhibited the highest accumulation in brain tissue among the 11 identified constituents of this formulation [17], suggesting its pivotal role in the therapeutic efficacy against cerebrovascular diseases, such as ischemic stroke. It contributes to the documented antioxidant, anti-inflammatory, and microcirculation-improving properties of these preparations [18]. Owing to its favorable bioactivity and structural characteristics, Pts can serve as a promising lead scaffold for further structural modification (Figure 1). Previous studies have reported a variety of Pts derivatives with diverse biological activities, including anti-cancer [19], anti-inflammatory [20], antioxidant, and neuroprotective effects [21]. These findings suggest that structural optimization of Pts is a feasible strategy for discovering novel anti-ischemic agents. Insight of the promising therapeutic efficacy of Pts in cerebrovascular diseases, this study was designed and screened a series of Pts hybrids via a bioactive substructure combination strategy (Table 1). This study aimed to develop novel anti-CIRI candidates with distinct mechanisms and favorable drug-like properties. In this study, we used an absorption, distribution, metabolism, excretion, and toxicity (ADMET) model to predict the drug likeness of this series of compounds. In addition, the functional regulatory effects of these compounds on BMECs and their antioxidant and anti-inflammatory effects under ischemia-reperfusion conditions were also studied. Finally, compound NO. **3** with significantly improved protective effects compared to Pts and was selected to investigate of its acute toxicity. Compound NO. **3** will be further explored for its mechanism of anti-ischemic stroke activity and may serve as a promising therapeutic candidate, broadening potential treatment options for CIRI.

## 2. Results

### 2.1. Synthesis of Candidates

Pterostilbene (purity > 98% by HPLC) was purchased from Shanghai Bide Pharmaceutical Technology Co., Ltd. (Shanghai, China). All the solvents and reagents were purchased from commercial sources and used as received. A small library of Pts derivatives was designed, and the structures are presented in Figure S1. Only the selected compounds used for further pharmacological evaluation were fully characterized (Figure S2).

**Scheme 1:**
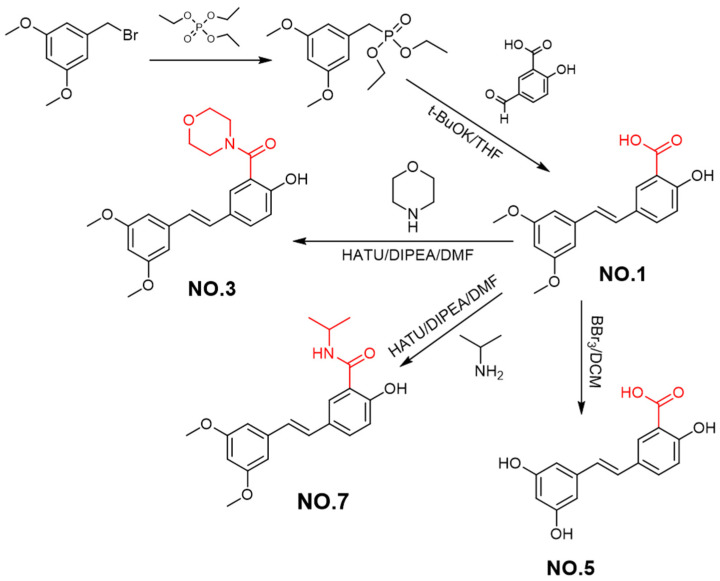
The synthetic route of derivatives NO. **1**, NO. **3**, NO. **5**, and NO. **7**. t-BuOK, potassium tert-butoxide; THF, tetrahydrofuran; HATU, 2-(7-Azabenzotriazol-1-yl)-N,N,N′,N′-tetramethyluronium hexafluorophosphate; DIPEA, N,N-Diisopropylethylamine; DMF, N,N-dimethylformamide; DCM, dichloromethane.

3,5-dimethoxybenzyl bromide was reacted with triethyl phosphate to prepare diethyl 3,5-dimethoxybenzyl phosphonate. The resulting phosphonate was then reacted with 5-formylsalicylic acid under basic conditions to afford a stilbene derivative. The synthetic routes of Pts derivatives (NO. **1**, NO. **3**, NO. **5**, and NO. **7**) are shown in Scheme 1.

#### 2.1.1. Synthesis of Compound NO. **1**

Diethyl 3,5-dimethoxybenzyl phosphonate (2.88 g, 0.010 mol) and t-BuOK (4.7 g, 0.042 mol) was dissolved in tetrahydrofuran (THF, 40 mL) under a nitrogen atmosphere and cooled to ~0 °C. A solution of 5-formylsalicylic acid (1.99 g, 0.012 mol) in THF (20 mL) was added dropwise to the cooled phosphonate solution. After completion of the addition, the reaction was maintained at 0 °C for 1 h and then allowed to warm to room temperature, where it was stirred for an additional 3 h. The reaction mixture was acidified with 1 N HCl to pH = 1–2 and extracted with ethyl acetate (EtOAc). The combined organic layers were washed with brine, dried over MgSO_4_, filtered, and concentrated under reduced pressure. Purification by flash column chromatography afforded compound NO. **1** as an off-white solid (0.96 g, 32%).

#### 2.1.2. Synthesis of Compound NO. **3**

Compound NO. **1** (0.30 g, 1 mmol), HATU (0.45 g, 0.012 mol), and N,N-diisopropylethylamine (DIPEA, 0.38 g, 0.03 mol) were dissolved in anhydrous DMF (15 mL) and stirred at room temperature for 12 h. Upon completion of the reaction, the mixture was acidified with 1 N HCl to pH = 1–2 and extracted with ethyl EtOAc. The combined organic layers were washed with brine, dried over MgSO_4_, filtered, and concentrated under reduced pressure. The crude product was purified by column chromatography to afford compound NO. **3** as an off-white solid (277 mg, 75% yield).

#### 2.1.3. Synthesis of Compound NO. **5**

Compound NO. **1** (150 mg, 0.5 mmol) was dissolved in anhydrous CH_2_Cl_2_ (15 mL) under nitrogen and cooled to −78 °C. A 1.0 M solution of BBr_3_ in CH_2_Cl_2_ (1.2 mmol) was added dropwise. The reaction mixture was allowed to warm to room temperature and stirred for 4 h. The reaction was quenched with ice water and extracted with CH_2_Cl_2_. The combined organic layers were washed with saturated NaHCO_3_ and brined, dried, and concentrated. Purification by silica gel column chromatography afforded compound C as a white solid (70.7 mg, 52%).

#### 2.1.4. Synthesis of Compound NO. **7**

Compound NO. **7**, a white solid compound, could be produced by a synthetic method similar to that used to prepare compound NO. **3** (52 mg, 78%).

### 2.2. Virtual Screening

A small library of Pts derivatives was initially screened. Full datasets of screening assays for the other derivatives are compiled in the Figures S3 and S4 and Table S1.

#### 2.2.1. Drug-Likeness Predicted by ADMET Analysis

**Figure 2:**
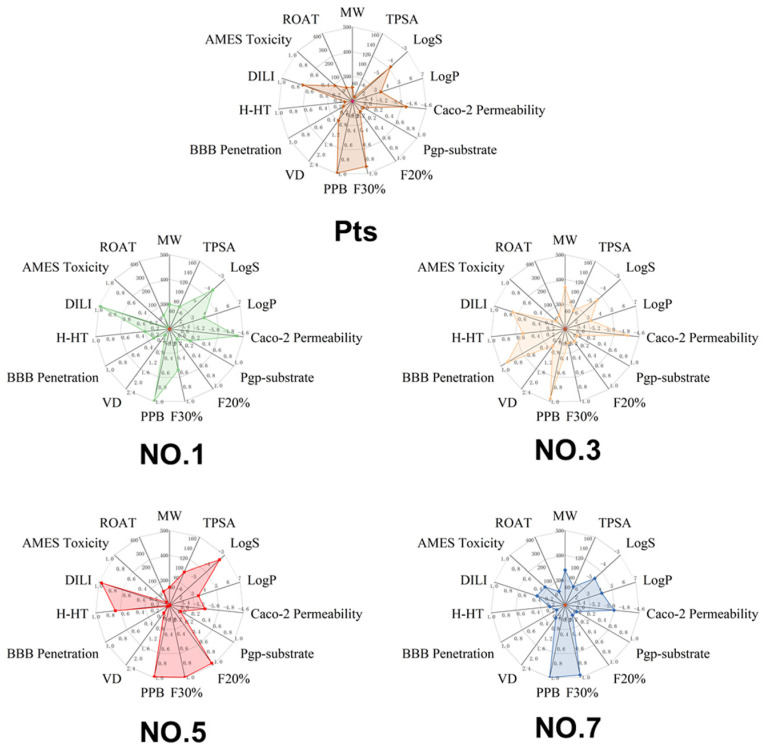
A radar plot displayed the key ADMET parameters of compounds predicted using ADMETlab 2.0. The reference ranges of each parameter were defined based on the actual predicted values across all compounds to comprehensively capture the distribution of the dataset. The evaluated parameters include MW (100–500 g/mol), TPSA (30–170 Å), LogS (from −7 to −3), LogP (1–7), Caco-2 permeability (from −5.6 to −4.6), VD (0–2.4 L/kg), as well as probability-based parameters (0–1), including P-gp substrate, F20%, F30%, PPB, BBB permeability, H-HT, DILI, AMES toxicity, and ROAT. MW, molecular weight; TPSA, topological polar surface area; PPB, plasma protein binding; VD, volume of distribution; H-HT, human hepatotoxicity; DILI, drug-induced liver injury; AMES, Ames mutagenicity; ROAT, rat oral acute toxicity.

The ADMET prediction is used for the initial assessment of drug pharmacological properties and for the preliminary screening of potential candidate drugs. The predicted ADMET parameters of the compounds are shown in Figure 2. Compounds with favorable drug-like properties are generally expected to comply with Lipinski’s rule of five [22]. ADMET prediction results indicated that all derivatives satisfied at least four of the Lipinski rules (MW ≤ 500 Da, n HA ≤ 10, n HD ≤ 5, Log P ≤ 5, n ROT ≤ 10), suggesting that these compounds possess acceptable drug-likeness and preliminary potential for oral administration. The human colon adenocarcinoma cell lines (Caco-2) have been widely used for evaluating the intestinal permeability of drugs due to their morphological and functional similarities [23]. The predicted Caco-2 permeability of compound NO. **5** (−5.293) suggested that it may have poor oral absorption. In the probability output values of P-glycoprotein (P-gp) substrates, all four compounds had a probability lower than 15%, indicating that these compounds were unlikely to be P-gp substrates, which may result in high oral bioavailability or improved central nervous system (CNS) permeability. Furthermore, Compounds NO. **3** exhibited favorable properties related to BBB penetration, with predicted probabilities approaching or exceeding 90% for achieving a brain-to-blood ratio greater than 10%. These results indicated that compounds NO. **3** may have the highest intestinal absorption capacity and BBB permeability and may have advantages in treating brain diseases.

#### 2.2.2. Brain or Intestinal Permeation Predicted by BOILED-EGG

**Figure 3:**
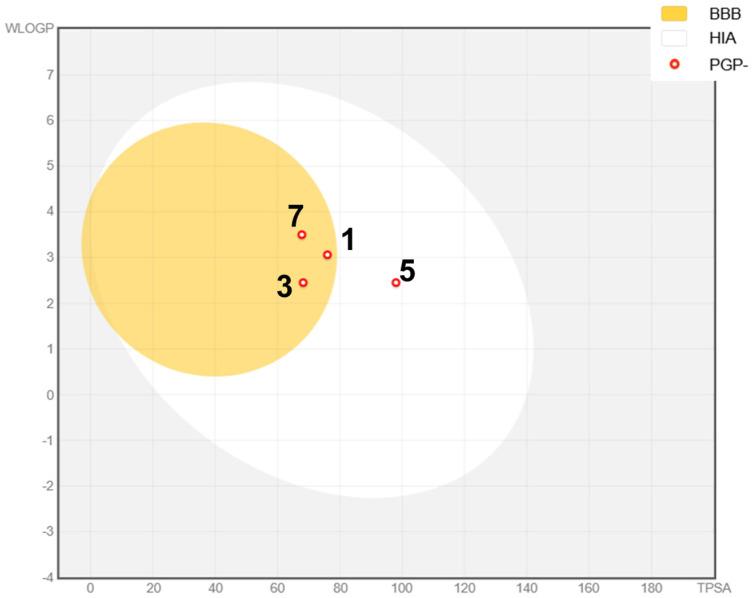
A BOILED-EGG model of Pts derivatives. The “yolk region” indicates a significant likelihood of BBB penetration, the “albumen region” indicates gastrointestinal absorption, and the “gray outer region” defines molecules characterized by limited brain absorption and penetration. The red points are expected to be a non-substrate of P-gp (PGP^−^). BBB, blood-brain barrier; HIA, human intestinal absorption; PGP, P-glycoprotein; WLOGP, Wildman-Crippen Log P; TPSA, topological polar surface area.

BOILED-EGG is used to estimate the ability of brain penetration, gastrointestinal absorption, and the possibility of P-gp substrate of a compound [24]. All 11 derivatives fell within the “albumen region” of the plot (Figure 3), indicating favorable intestinal absorption properties. Notably, compounds NO. **1**, NO. **3**, and NO. **7** were located in the “yolk zone”, indicating favorable BBB permeability. In contrast, NO. **5** showed a relative low BBB penetration efficiency.

Consequently, based on the results of the two virtual model screening experiments, NO. **3** is the most promising candidate molecule for treating cerebral ischemia.

### 2.3. Biological Activities Evaluation In Vitro

#### 2.3.1. Effects of the Derivatives on Cell Viability Under Oxygen-Glucose Deprivation/Reperfusion (OGD/R) Injury Models

**Figure 4:**
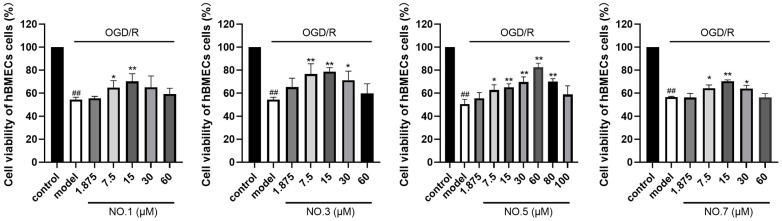
Effects of derivatives on OGD/R-induced hBMECs injury models after 24 h co-incubation. Data are presented as The Mean ± SD of each group (*n* = 3). ^##^ *p* < 0.01 vs. control group, * *p* < 0.05, ** *p* < 0.01 vs. model group. OGD/R, oxygen-glucose deprivation/reperfusion.

To comprehensively evaluate the in vitro biological activity of the derivatives, hBMECs and SH-SY5Y cells were selected to assess their effects at the neuronal and endothelial levels. As illustrated in Figure 4, the cell viability of OGD/R-induced hBMECs was significantly reduced compared to the control group (*p* < 0.01). Treatment with compounds NO. **6**, NO. **8**, NO. **9**, NO. **10**, and NO. **11** for 24 h did not result in a significant increase in cell viability (*p* > 0.05), whereas compounds NO. **2** and NO. **4** significantly enhanced cell viability at concentrations of 0.94–3.75 µM (Figure S5). In contrast, hBMECs treated with NO. **1**, NO. **3**, NO. **5**, and NO. **7** at specific concentrations for 24 h markedly increased cell viability. The effective concentrations of these compounds ranged from 1.875 to 80 μM and exhibited an obvious bell-shaped dose–response relationship.

**Figure 5:**
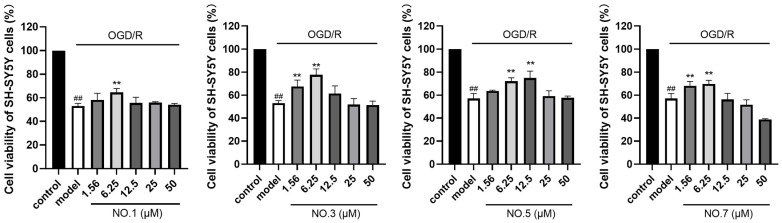
Effects of derivatives on OGD/R-induced SH-SY5Y injury models after 24 h co-incubation. Data are presented as the Mean ± SD of each group (*n* = 3). ^##^ *p* < 0.01 vs. control group, ** *p* < 0.01 vs. model group. OGD/R, oxygen-glucose deprivation/reperfusion.

Under the same conditions, experiments on the protective effects of the derivatives on SH-SY5Y cells after OGD/R were conducted, and the results are shown in Figure 5. The cell viability of SH-SY5Y cells significantly decreased after OGD/R treatment (*p* < 0.01). Treatment with compounds NO. **2**, NO. **6**, NO. **8**, NO. **9**, and NO. **11** for 24 h did not result in a significant increase in cell viability (*p* > 0.05), whereas compounds NO. **4** and NO. **10** significantly enhanced cell viability at concentrations of 6.25–25 µM (Figure S6). From the range of 1.56 to 50 µM, compounds NO. **1**, NO. **3**, NO. **5**, and NO. **7**, co-incubated with the cells for 24 h, could significantly increase the cell survival rate (*p* < 0.05).

The maximal recovery rate was further evaluated, and the sensitivity of different cell lines to the derivatives was assessed. Table S2 shows the sensitivity of compounds NO. **2**, NO. **4**, and NO. **10** to different cell lines. Table 2 showed that the response trends of the two cell models to most compounds were basically the same, but hBMECs showed higher sensitivity or responsiveness to the specific compounds. Given the crucial position of hBMECs in BBB injury, oxidative stress, and inflammation in OGD/R models, subsequent experiments focused on hBMECs.

Based on the results of MTT assays, compounds NO. **1**, NO. **3**, NO. **4**, NO. **5**, and NO. **7** all increased the survival rates of both the SH-SY5Y and hBMECs cell models after OGD/R. As hBMECs were selected as the primary focus for subsequent experiments, compounds NO. **1**, NO. **2**, NO. **3**, NO. **4**, NO. **5**, and NO. **7**, which significantly improved cell viability in OGD/R induced-hBMECs, were chosen for further investigation.

#### 2.3.2. TI-Based Assessment of the Derivatives

**Figure 6:**
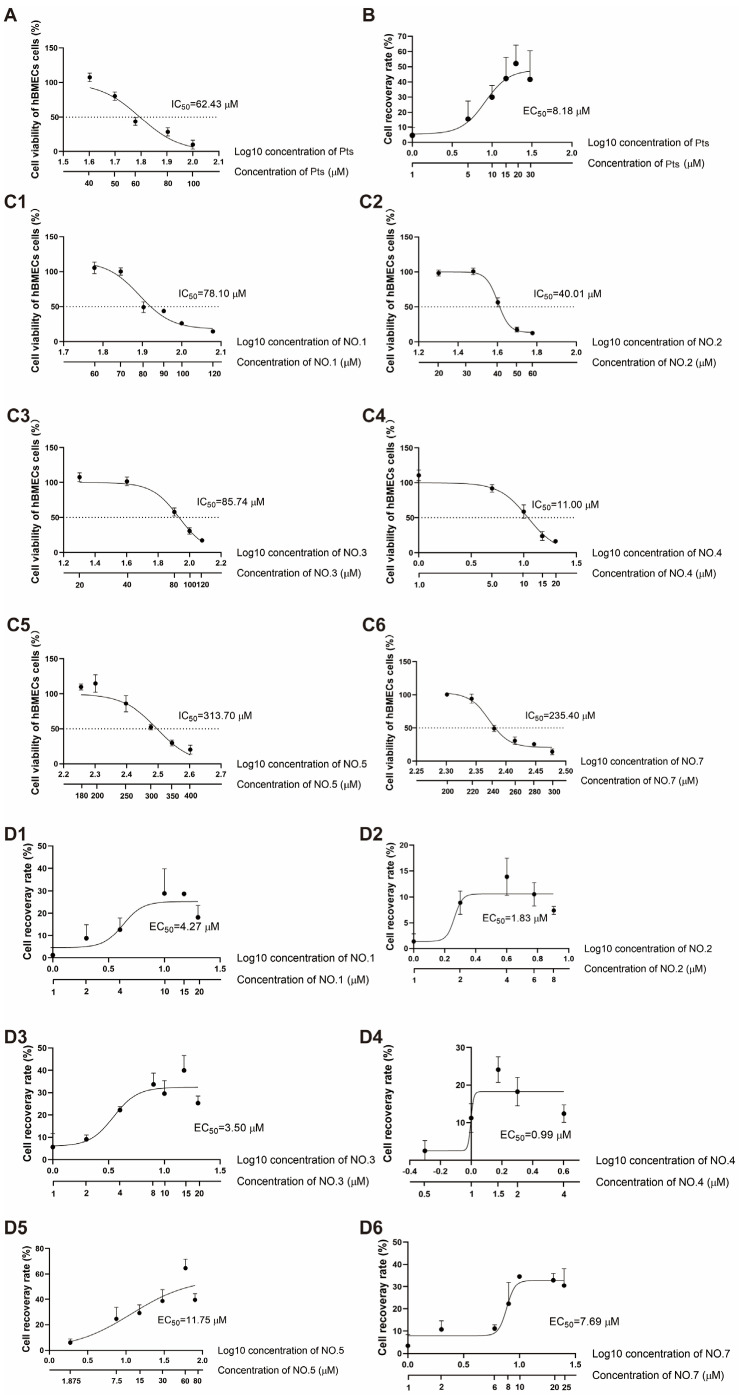
Effects of positive control Pts and its derivatives on normal and OGD/R-induced hBMECs injury models after 24 h co-incubation. (**A**) The IC_50_ value of Pts’ effect on normal hBMECs for 24 h. Data are presented as the Mean ± SD of each group (n = 5). (**B**) The EC_50_ value of Pts’ effect on OGD/R-induced hBMECs for 24 h. Data are presented as the Mean ± SD of each group (n = 5). (**C1**–**C6**) The IC_50_ value of the derivatives’ effect on normal hBMECs for 24 h. Data are presented as Mean ± SD of each group (NO. **1**, NO. **3**, n = 5; NO. **5**, NO. **7**, n = 3). (**C1**–**C6)** represented the data of NO. **1**, NO. **3**, NO. **5**, and NO. **7**, respectively. (**D1**–**D6**) The EC_50_ value of the derivatives’ effect on OGD/R-induced hBMECs for 24 h. Data are presented as the Mean ± SD of each group (n = 3). (**D1**–**D6**) represented the data of NO. **1**, NO. **3**, NO. **5**, and NO. **7**, respectively. IC_50_, half inhibition concentration; EC_50_, half effective concentration.

The effective concentration and the maximal effective concentration vary in different compounds. To circumvent this limitation, the therapeutic index (TI) was calculated as a normalized metric, enabling comparison of both potency and efficacy between derivatives and Pts (Table 3). We detected the IC_50_ values (half inhibition concentration) of the derivatives to normal hBMECs and the EC_50_ values (half effective concentration) under OGD/R injury conditions (Figure 6). All four candidate compounds had greater TI than Pts. However, due to the lowest maximal recovery rate of NO. **2** (13.89%) and the highest cytotoxicity of NO. **4** (IC_50_ = 11.00 μM), these two compounds were excluded (Figure S7, Table S3). Thus, compounds NO. **1**, NO. **3**, NO. **5**, and NO. **7** were selected for further investigation. 

The EC_50_ values of the selected compounds ranged from 3.50 to 11.75 μM, and 7.5 μM falls within this effective concentration range. At this concentration, all compounds exhibited significant biological activity while remaining well below their respective IC_50_ values, indicating low cytotoxicity (Figure 4). This concentration lies within the dynamic range of the dose–response curve, where changes in drug concentration result in pronounced biological effects. Therefore, this concentration was used in subsequent experiments.

#### 2.3.3. Compounds NO. **1**, NO. **3**, NO. **5** and NO. **7** Reduced Lactate Dehydrogenase (LDH) Release Under OGD/R Injury hBMECs

**Figure 7:**
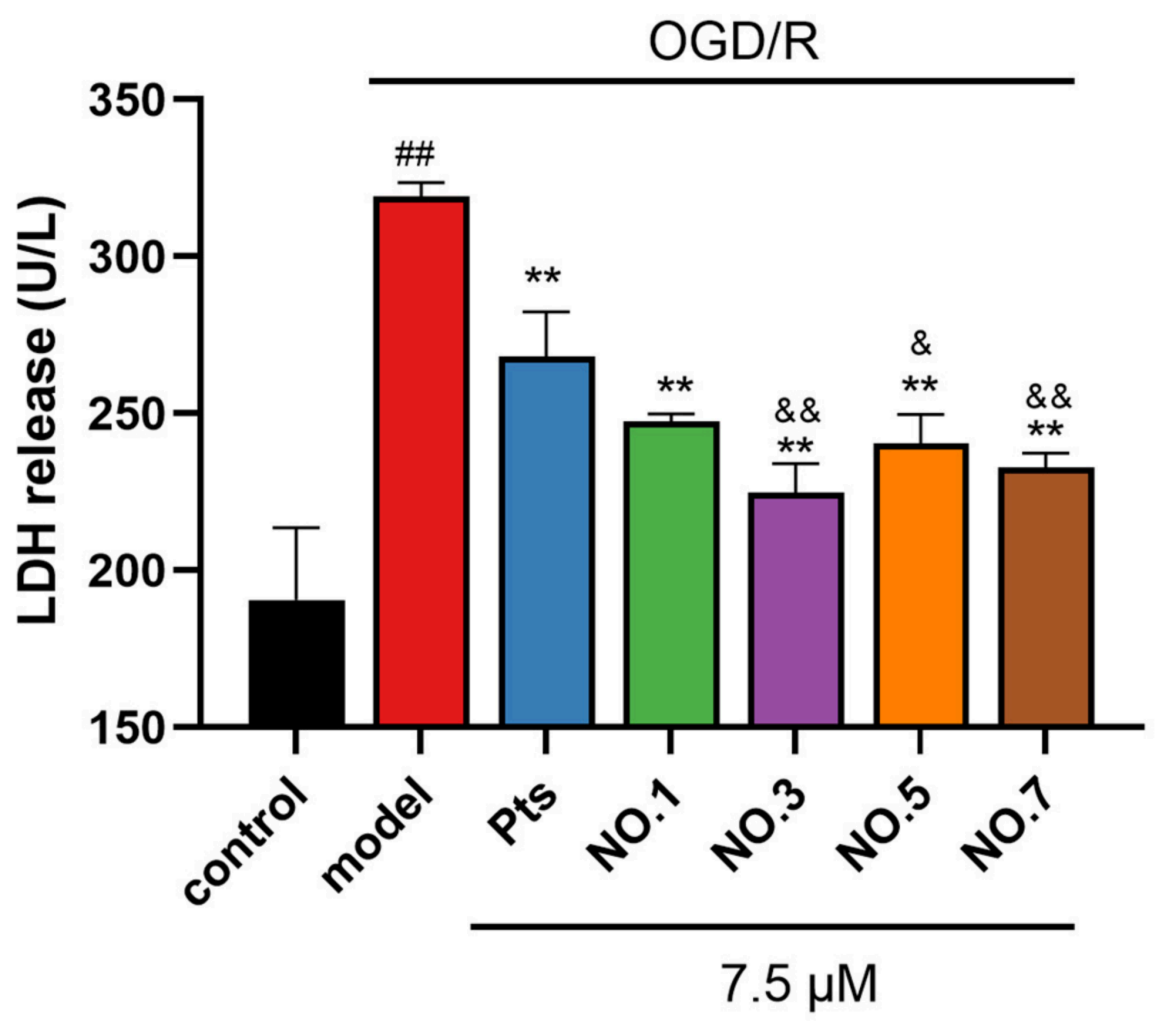
Effects of positive control Pts and NO. **1**, NO. **3**, NO. **5** and NO. **7** on the LDH release of OGD/R-induced hBMECs. Data are presented as the Mean ± SD of each group (n = 5). ^##^ *p* < 0.01 vs. control group, ** *p* < 0.01 vs. model group, ^&^ *p* < 0.05, ^&&^ *p* < 0.01 vs. Pts group. LDH, lactate dehydrogenase.

LDH release was an indirect indicator of cellular injury. As depicted in Figure 7, LDH levels in the supernatant of OGD/R injury hBMECs were significantly increased (*p* < 0.01). Treatment with the positive control Pts and all four candidate compounds markedly reduced LDH release (*p* < 0.01). Compared with the model group, Pts, NO. **1**, NO. **3**, NO. **5**, and NO. **7** reduced LDH release by 16.06%, 22.48%, 29.62%, 24.69%, and 27.05%, respectively. Notably, NO. **3**, NO. **5**, and NO. **7** exhibited stronger inhibitory effects on LDH release than Pts, with statistically significant differences (NO. **5**, *p* < 0.05; NO. **3**, NO. **7**, *p* < 0.01). Their effects were 1.84, 1.54, and 1.68 times that of Pts, respectively. This result showed that, under the condition of OGD/R injury hBMECs, compounds NO. **3**, NO. **5**, and NO. **7** manifested superior cytoprotective effect compared with the prototype drug Pts and may more effectively maintain the integrity of the cell membrane.

#### 2.3.4. Compounds NO. **1**, NO. **3**, NO. **5** and NO. **7** Promoted Cell Survival, Migration, and Angiogenesis Under OGD/R Injury hBMECs

**Figure 8:**
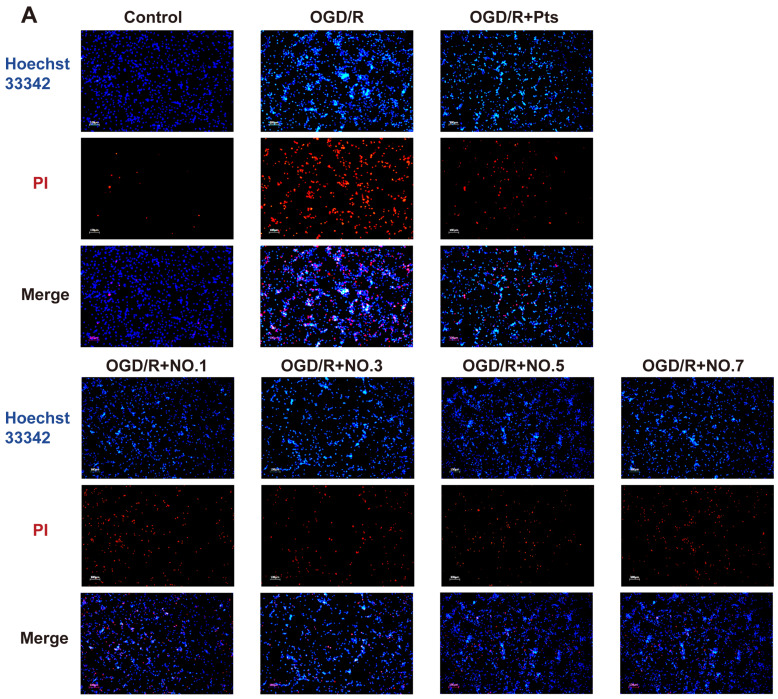

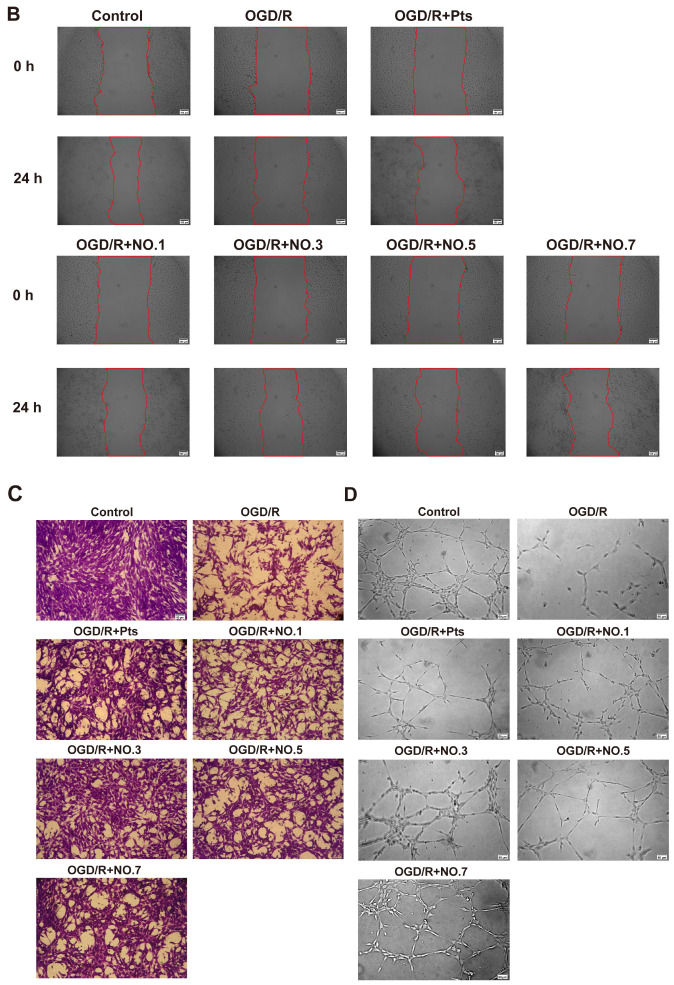

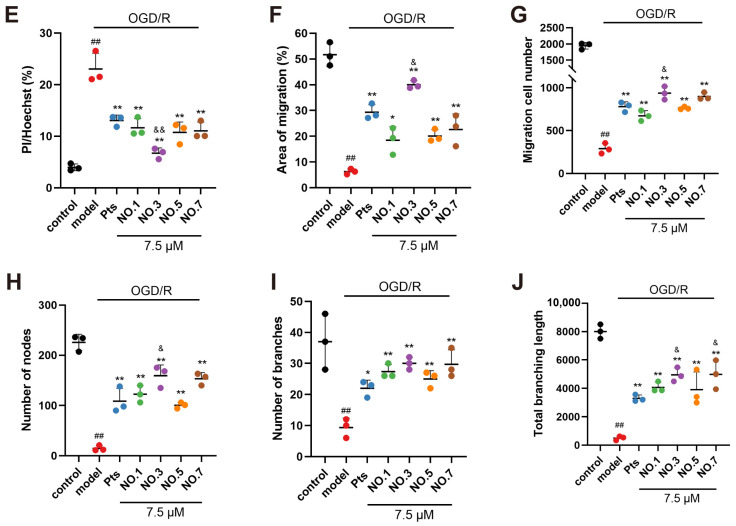
Positive control Pts and NO. **1**, NO. **3**, NO. **5**, and NO. **7** recovered the ability of proliferation, migration, and angiogenesis of OGD/R-injured hBMECs. (**A**) OGD/R-injured hBMECs were treated with Pts, NO. **1**, NO. **3**, NO. **5**, and NO. **7** (7.5 µM) for 24 h and then stained with Hoechst 33342/PI for fluorescence microscopy analysis (×100, Scale Bar = 100 µm). (**B**) Images obtained from the hBMECs scratch assay after 24 h drug incubation (7.5 µM) following OGD/R injury (×100, Scale Bar = 100 µm). (**C**) Images of the hBMECs Transwell migration assay for 6 h (×100, Scale Bar = 100 µm). (**D**) Images of the hBMECs tube formation assay for 6 h (×200, Scale Bar = 50 µm). (**E**) The ratio of PI/Hoechst (%) after stained. (**F**) Quantitative analysis of migration rate in the hBMECs scratch assay. (**G**) Quantitative analysis of migration cell numbers in the hBMECs Transwell migration assay. (**H**–**J**) Quantitative analysis of the numbers in the nodes, branches, and total branching length in the hBMECs tube formation assay. Data are presented as the Mean ± SD of each group (n = 3). ^##^ *p* < 0.01 vs. control group, * *p* < 0.05, ** *p* < 0.01 vs. model group, ^&^ *p* < 0.05, ^&&^ *p* < 0.01 vs. Pts group.

The effects of the derivatives on cell survival, migration, and angiogenesis were evaluated, as BMECs have an important position in maintaining BBB integrity and cerebral perfusion. OGD/R induced apoptosis in hBMECs and significantly impaired both horizontal and vertical migratory capacities, as well as tube formation ability. Quantitative analysis of PI/Hoechst fluorescence intensity (Figure 8A,E) demonstrated that NO. **3** exerted a stronger anti-apoptotic effect than Pts (*p* < 0.01). In the scratch assay (Figure 8B,F), taking the positive control Pts as the standard, the relative effects of NO. **1**, NO. **3**, NO. **5**, and NO. **7** on the area of migration of OGD/R-injured hBMECs were 62.91%, 136.52%, 68.49%, and 77.25%, respectively, and 86.22%, 120.28%, 97.77%, and 115.32% in Transwell migration assay (Figure 8C–G). NO. **3** could effectively promote both horizontal and vertical migration ability, and there was a statistically significant difference compared with Pts. The results of the tube formation assay (Figure 8D,H–J) were consistent with these results, and NO. **3** manifested the optimal effects. These results indicated that compounds NO. **1**, NO. **3**, NO. **5**, and NO. **7** could all improve the abilities of cell survival, migration, and angiogenesis in OGD/R injury hBMECs, and NO. **3** demonstrates better biological activities compared to Pts.

#### 2.3.5. Compounds NO. **1**, NO. **3**, NO. **5** and NO. **7** Improved Antioxidant Capacity Under OGD/R Injury hBMECs

**Figure 9:**
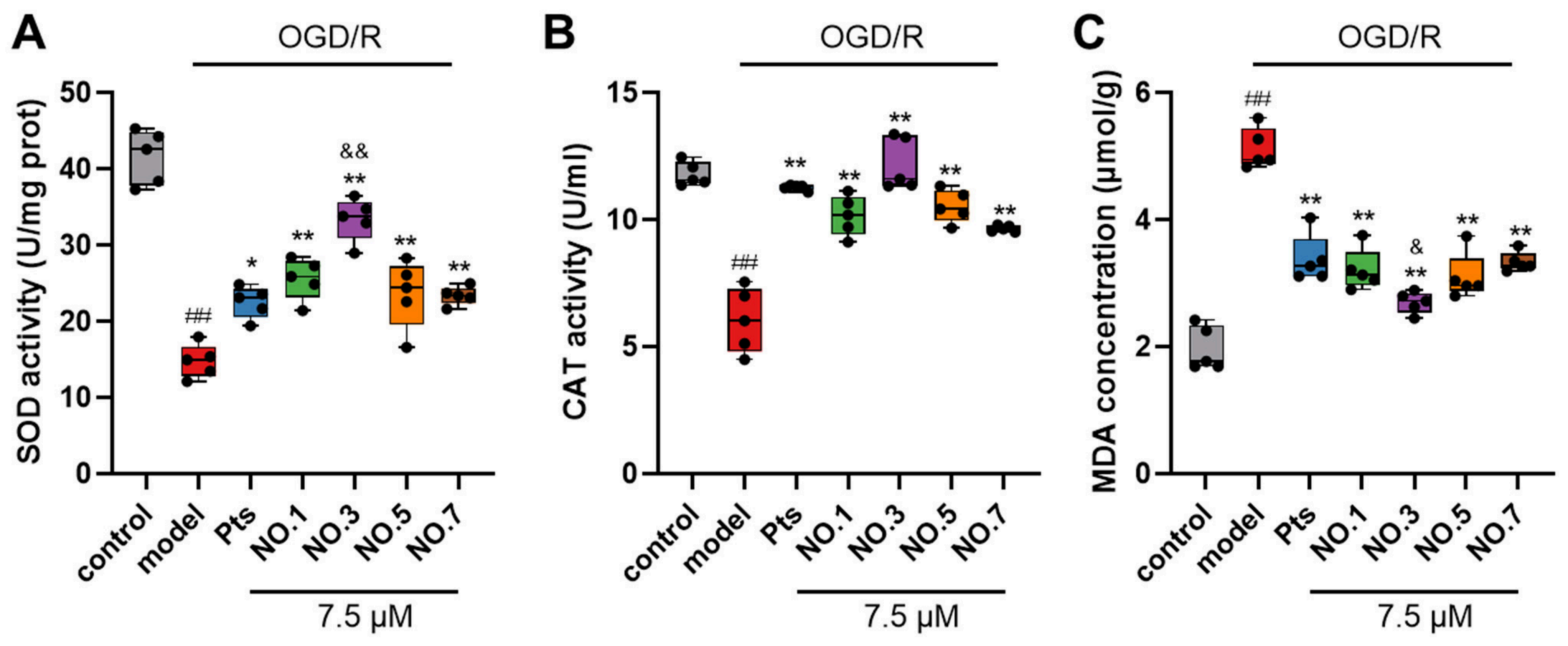
Positive control Pts and NO. **1**, NO. **3**, NO. **5**, and NO. **7** alleviated the oxidative stress of OGD/R-induced hBMECs. (**A**–**C**) SOD, CAT, and MDA levels in hBMECs. Data are presented as the Mean ± SD of each group (n = 5). ^##^ *p* < 0.01 vs. control group, * *p* < 0.05, ** *p* < 0.01 vs. model group, ^&^ *p* < 0.05, ^&&^ *p* < 0.01 vs. Pts group. SOD, superoxide dismutase; CAT, catalase; MDA, malondialdehyde.

OGD/R is associated with elevated oxidative stress, characterized by increased oxidative products and decreased antioxidant enzyme levels [25]. Following OGD/R, superoxide dismutase (SOD) and catalase (CAT) concentrations decreased, while malondialdehyde (MDA) levels increased significantly (Figure 9), indicating hBMECs were in an oxidative stress state. In contrast, compared with the model group, treatment with NO. **1**, NO. **3**, NO. **5**, NO. **7**, or Pts upregulated the levels of SOD and CAT and downregulated the level of MDA, with NO. **3** showing the most significant improvement on SOD and MDA compared with the Pts group (*p* < 0.01). Among the derivatives, compound NO. **3** exhibited the strongest regulatory effects on SOD, CAT, and MDA levels in vitro, which were approximately 2.40, 1.17, and 1.39 times greater than those of Pts, respectively.

Collectively, these results indicate that compounds NO. **1**, NO. **3**, NO. **5**, and NO. **7** significantly protected hBMECs against OGD/R-induced injury and displayed higher TI than the prototype compound Pts. All four compounds reduced LDH release, restored migration and angiogenic capacity, and attenuated oxidative stress in vitro. Notably, NO. **3** exhibited superior activity relative to Pts. Although virtual screening suggested that NO. **3** might be the most promising candidate, the potential in vivo efficacy of compounds NO. **1**, NO. **5**, and NO. **7** could not be excluded. Therefore, all four compounds were advanced to subsequent in vivo evaluation.

### 2.4. Biological Evaluation of Derivatives In Vivo

#### 2.4.1. Effects of the Derivatives on the Body Weight and Survival Rate of Middle Cerebral Artery Occlusion/Reperfusion (MCAO/R) Rats

**Figure 10:**
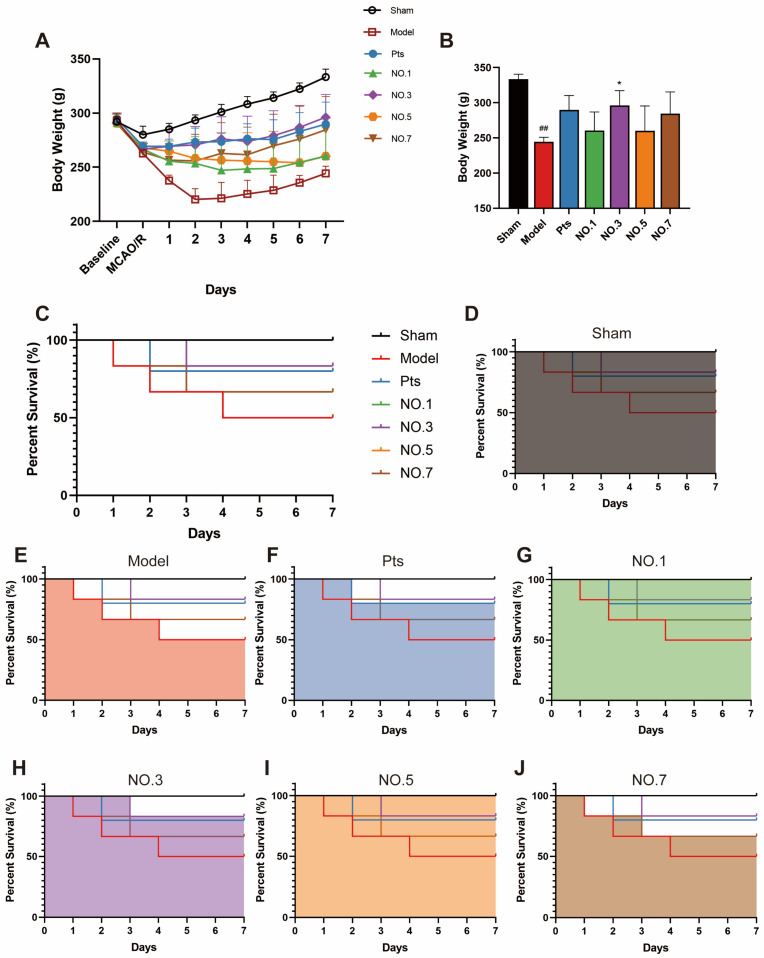
The effects of the positive control Pts and NO. **1**, NO. **3**, NO. **5**, and NO. **7** on the body weight and survival rate in MCAO/R rats. (**A**) The body weight of the rats in each group. (**B**) The body weight of the rats in each group on the 7th day after administration. Data are presented as the Mean ± SD of each group (model, n = 3; Pts and NO. **7**, n = 4; other groups, n = 5). ^##^ *p* < 0.01 vs. control group, * *p* < 0.05 vs. model group. (**C**) Summary of survival rates during the 7-day observation period. (**D**–**J**) The survival rate of rats in each group during the 7-day observation period.

The rats were continuously administered the drug for 7 days after the MCAO/R, and the body weight were recorded everyday (Figure 10A,B). Two days after the operation, the body weight of MCAO/R rats continued to decrease and gradually recovered on the third day. The treatment with Pts and the derivatives could increase the body weight of MCAO rats to a certain extent. The effect of increasing body weight of NO. **3** was the strongest, and there was a statistically significant difference compared with the model group (*p* < 0.05).

The survival rates of each group were recorded. As shown in Figure 10B–I, the first 3 days were the acute phase of MCAO/R, and the survival rate of rats tended to stabilize after 3 days. The model group exhibited the highest mortality following MCAO/R. Compared with the model group, all treatment groups showed a reduced mortality rate after MCAO/R. Among them, the NO. **1** and NO. **5** groups exhibited the lowest mortality, with no deaths observed during the experimental period. The Pts, NO. **3**, and NO. **7** groups were observed to have partial mortality. In terms of increasing survival rate, the effects of Pts, NO. **3**, and NO. **7** were similar in the acute phase (the first 3 days after surgery). The efficacies of the NO. **1** and NO. **5** groups were better than the Pts group under the experimental conditions (5 mg/kg, ip., 7 d).

#### 2.4.2. Effects of the Derivatives on Cerebral Blood Flow (CBF) and Infarct Volume of MCAO/R Rats

**Figure 11:**
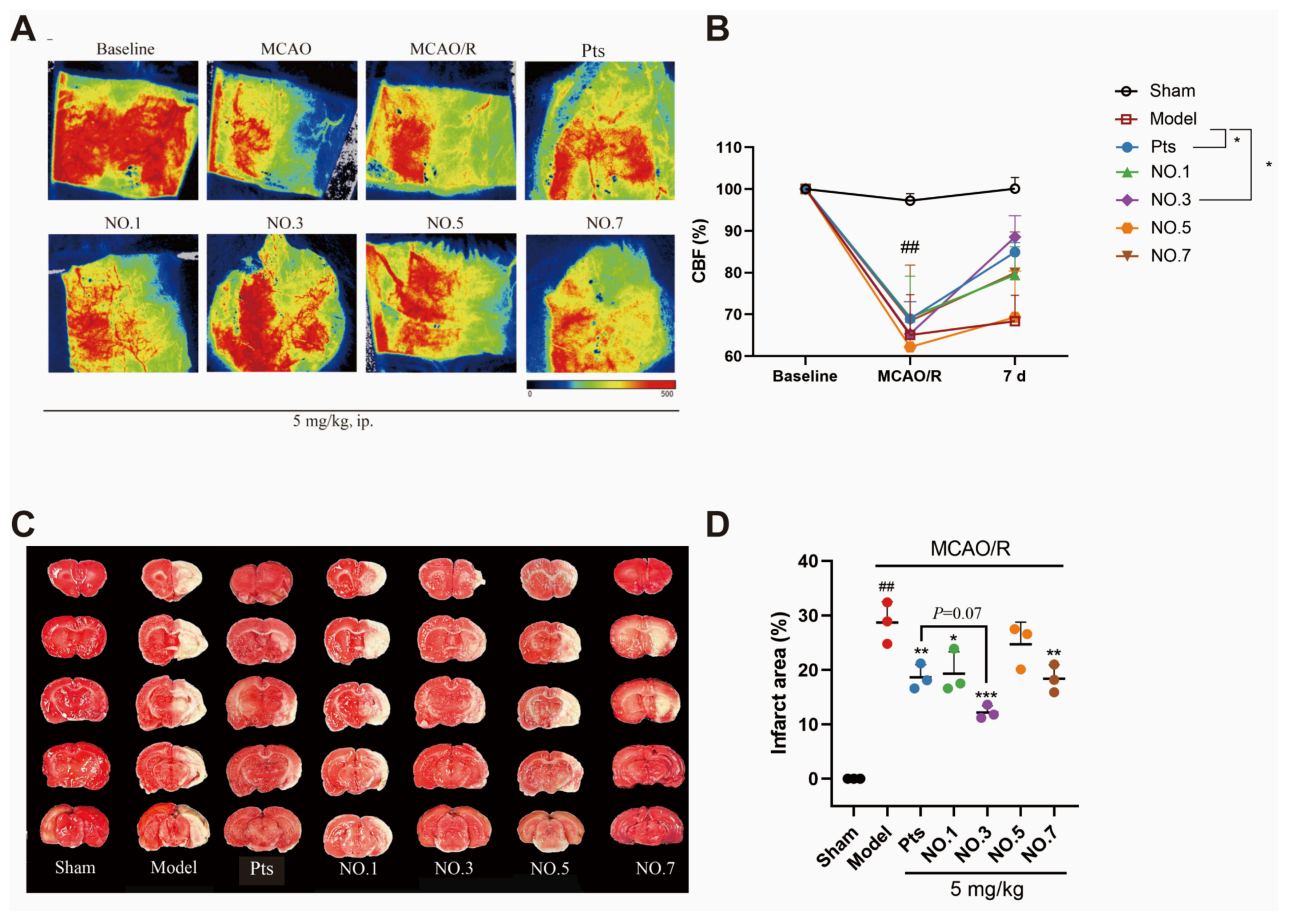
The effects of the positive control Pts and NO. **1**, NO. **3**, NO. **5**, and NO. **7** on CBF and infarct volume. (**A**) Representative images of the CBF in each group at MCAO/R and 7 days after reperfusion. (**B**) Quantitative analysis of the CBF in each group (model, n = 3; Pts and NO. **7**, n = 4; other groups, n = 5). (**C**) Representative images of the TTC staining in each group after 7 days treatment (n = 3). (**D**) Quantitative analysis of the infarct volume in each group (n = 3). Data are presented as the Mean ± SD of each group. ^##^ *p* < 0.01 vs. control group, * *p* < 0.05, ** *p* < 0.01, *** *p* < 0.001 vs. model group. CBF, cerebral blood flow.

To evaluate microcirculatory recovery, CBF changes were monitored by laser speckle imaging during ischemia and reperfusion. Following MCAO/R surgery, CBF decreased to approximately 60% of baseline in all groups (Figure 11A,B). After 7 days of treatment, CBF recovery varied among groups: model (5.07%), Pts (23.10%), NO. **1** (14.70%), NO. **3** (35.48%), NO. **5** (11.48%), and NO. **7** (16.77%). NO. **3** demonstrated the greatest CBF recovery, and it was 1.22 times that of Pts. These results indicated that NO. **3** enhanced the recovery of CBF, and NO. **1** and NO. **7** showed a tendency to increase CBF after 7 days of treatment of MCAO/R rats.

The TTC staining method was used to evaluate the cerebral infarction rate of each group (Figure 11C,D). The cerebral infarction rate of MCAO/R rats (28.70 ± 3.80) was statistically different compared with the sham group (*p* < 0.01). After 7 days of treatment, Pts (18.63 ± 2.35%), NO. **1** (19.33 ± 3.98%), NO. **3** (12.20 ± 1.25%), and NO. **7** (18.37 ± 2.55%) significantly reduced cerebral infarct volume compared with the model group (NO. **1**, *p* < 0.05; other groups, *p* < 0.01). NO. **3** showed the strongest effect, reducing infarct volume by 57.49% (of the model group) and achieving 34.51% lower infarct volume than Pts (*p* = 0.07). NO. **1** and NO. **7** exhibited comparable efficacies to Pts, while NO. **5** (24.73 ± 4.04%) showed no significant difference from the model group. These results indicated that NO. **1**, NO. **3**, and NO. **7** had a good effect on the reduction of cerebral infarction volume in 7 days after MCAO/R surgery, NO. **3** was better than that of Pts, and there was a trend between the two compounds.

#### 2.4.3. Effect of the Derivatives on Neurological Function of MCAO/R Rats

**Figure 12:**
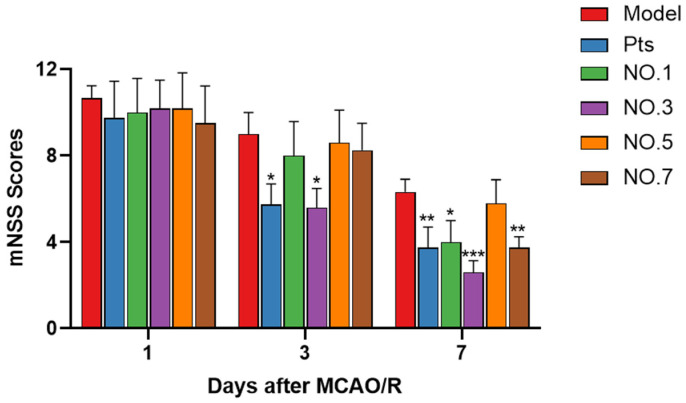
The mNSS of MCAO/R rats 1, 3, and 7 days after MCAO surgery in each group. Data are presented as the Mean ± SD of each group (model, n = 3; Pts and NO. **7**, n = 4; other groups, n = 5). * *p* < 0.05, ** *p* < 0.01, *** *p* < 0.001 vs. model group. mNSS, modified neurological severity score.

To evaluate the effect of the derivatives on the neurological function of MCAO/R rats, mNSS scores were assessed at **1**, **3**, and **7** days (Figure 12). At 24 h, all groups exhibited moderate to severe deficits. By day 3, Pts (5.75 ± 0.96) and NO. **3** (5.60 ± 0.89) significantly reduced neurological deficit scores compared with model (9.00 ± 1.00), indicating that both Pts and NO. **3** exerted early neuroprotection. At day 7, Pts (3.75 ± 0.96), NO. **1** (4.00 ± 1.00), NO. **3** (2.60 ± 0.55), and NO. **7** (3.75 ± 0.50) showed marked improvements (40.79%, 36.84%, 58.95%, and 40.79% reduction of the model group, respectively), while NO. **5** (5.80 ± 1.09) showed no significant difference compared to the model (6.33 ± 0.58). These results suggested that the administration of NO. **1**, NO. **3**, and NO. **7** could significantly improve neurological deficits, and NO. **3** demonstrated superior efficacy (1.45 times of Pts), whereas the effects of NO. **1** and NO. **7** were comparable to Pts (0.90 and 1.00 times of Pts).

#### 2.4.4. Effect of the Derivatives on Cellular Damage of MCAO/R Rats

**Figure 13:**
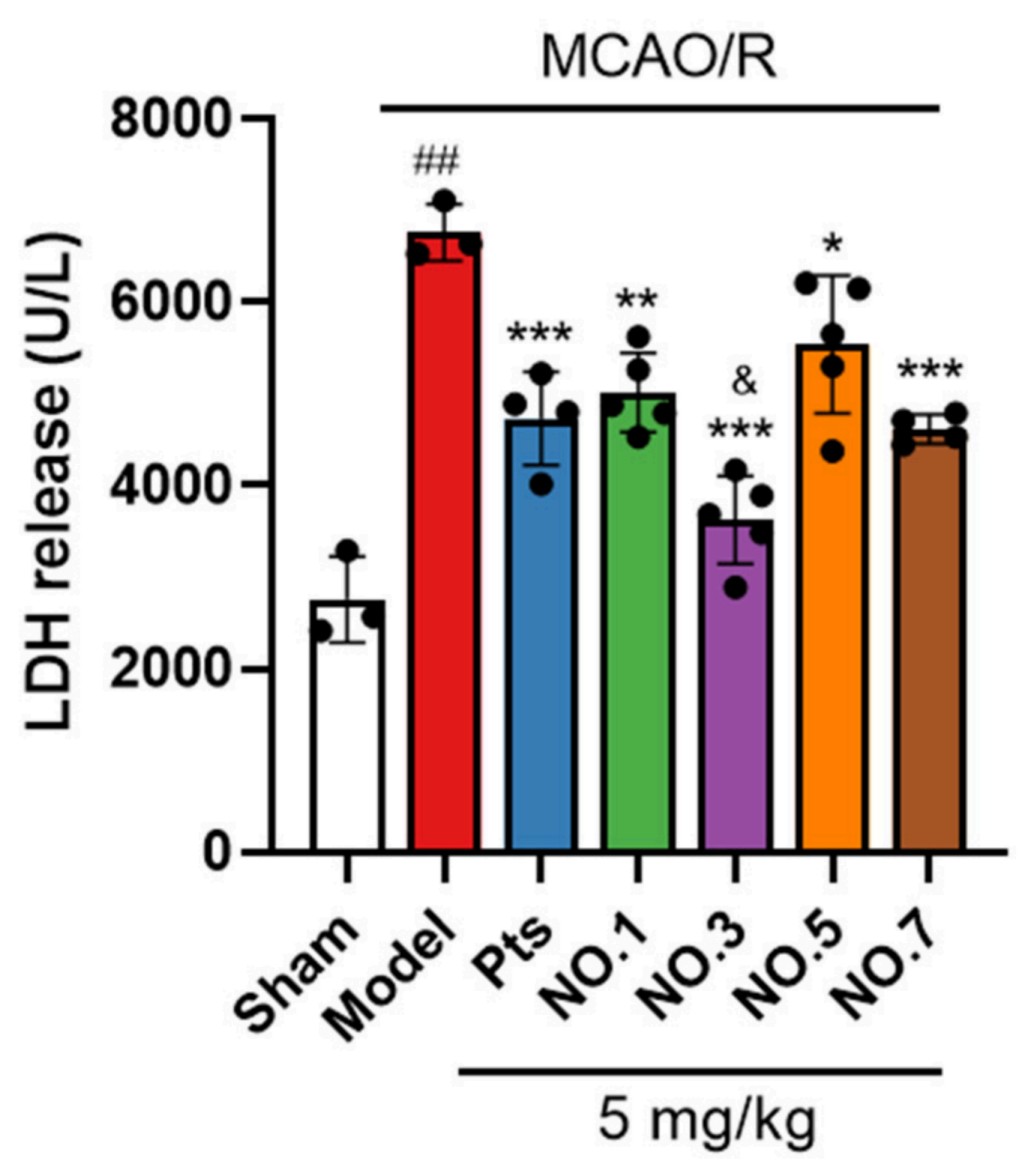
The effects of the positive control Pts and NO. **1**, NO. **3**, NO. **5**, and NO. **7** on serum LDH level. Quantitative analysis of the serum LDH level in each group. Data are presented as the Mean ± SD of each group (sham, model, n = 3; Pts and NO. **7**, n = 4; other groups, n = 5). ^##^ *p* < 0.01 vs. control group, * *p* < 0.05, ** *p* < 0.01, *** *p* < 0.001 vs. model group, ^&^ *p* < 0.05 vs. Pts group. LDH, lactate dehydrogenase.

After continuous administration for 7 days, the LDH content in the serum of rats in each group was detected, and the results were presented in Figure 13. The level of LDH in the serum of rats in the model group increased by 144.96% compared with that in the sham group (*p* < 0.01). Treatment of Pts, NO. **1**, NO. **3**, NO. **5**, and NO. **7** significantly downregulated LDH level. Compared with the model group, the contents of LDH decreased by 29.98%, 25.78%, 46.41%, 18.05%, and 31.68%, respectively. Among them, the LDH level in the serum of rats in NO. **3** group was the lowest, and there was a statistically significant difference compared with the Pts group (*p* < 0.05). Numerically, the LDH level in the serum of rats in NO. **3** group was 76.54% of that in the Pts group. These results showed that all four derivatives could inhibit the release of LDH in vivo.

#### 2.4.5. Effect of the Derivatives on Oxidative Stress Markers and Inflammatory Cytokines in the Serum of MCAO/R Rats

**Figure 14:**
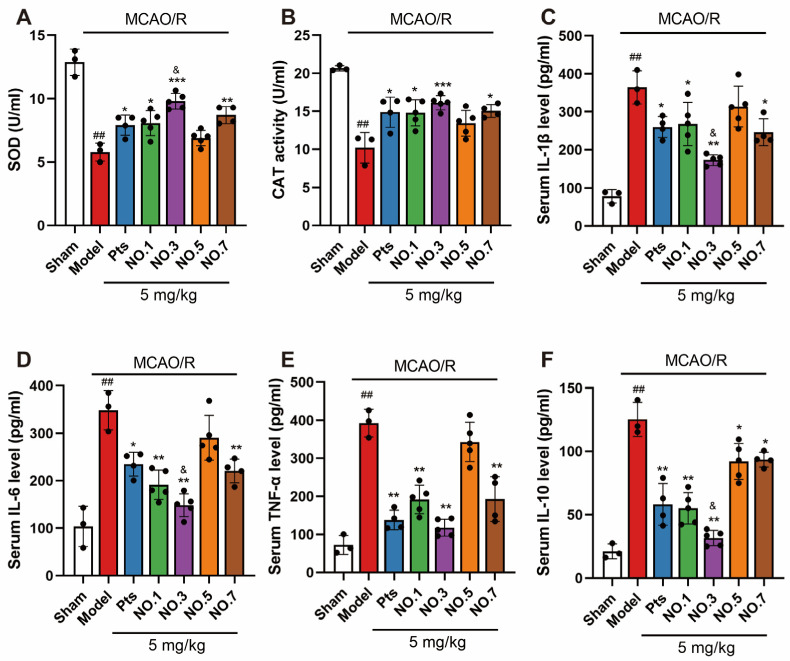
The effects of the positive control Pts and NO. **1**, NO. **3**, NO. **5**, and NO. **7** on serum oxidative stress markers and inflammatory cytokines. (**A**,**B**) Quantitative analysis of serum SOD, CAT level in each group. (**C**–**F**) Quantitative analysis of serum pro-inflammatory cytokines IL-1β, IL-6, and TNF-α and anti-inflammatory cytokines IL-10 level in each group. Data are presented as the Mean ± SD of each group (sham and model, n = 3; Pts and NO. **7**, n = 4; other groups, n = 5). ^##^ *p* < 0.01 vs. control group, * *p* < 0.05, ** *p* < 0.01, *** *p* < 0.001 vs. model group, ^&^ *p* < 0.05 vs. Pts group. SOD, superoxide dismutase; CAT, catalase; IL-1β, interleukin-1β; IL-6, interleukin-6; IL-10, interleukin-10; TNF-α, tumor necrosis factor-α.

As shown in Figure 14A,B, serum SOD and CAT levels were markedly reduced in the model group compared with the sham group (*p* < 0.01). After administration for 7 days, the Pts, NO. **1**, NO. **3**, and NO. **7** all had significantly increased the serum SOD and CAT content (*p* < 0.01). NO. **3** demonstrated superior antioxidant activity, increasing the SOD and CAT by 69.62% and 57.86% in the model group, with efficacies approximately 1.89 and 1.27 times greater than Pts, respectively.

The effects of derivatives on the inflammatory response induced by MCAO/R were evaluated by detecting the levels of pro-inflammatory factors IL-1β, IL-6, and TNF-α (Figure 14C–E) and anti-inflammatory factor IL-10 (Figure 14F) in rat serum. The levels of pro-inflammatory factors IL-1β, IL-6, and TNF-α in the serum of model group rats were significantly increased compared with the sham group (*p* < 0.01). Continuous treatment for 7 days with compounds Pts and NO. **1**, NO. **3**, NO. **5**, and NO. **7** reduced pro-inflammatory cytokine levels to varying extents. The content of anti-inflammatory factor IL-10 in the serum of model group rats was significantly increased (*p* < 0.01), suggesting that the anti-inflammatory effect was initiated in the MCAO/R rats. Administration of the same dose of Pts, NO. **1**, NO. **3**, NO. **5**, and NO. **7** for 7 consecutive days could reduce the level of IL-10, and all of them showed statistical differences compared with the model group (*p* < 0.05 or *p* < 0.01). Among them, NO. **3** exhibited the most potent anti-inflammation effects, as evidenced by 52.56%, 57.43%, 70.07%, and 74.73% decreases in IL-1β, IL-6, TNF-α, and IL-10 levels (1.83, 1.76, 1.08, and 1.40 times that of Pts), respectively, compared with the model group.

Taken together, compounds NO. **1** and NO. **7** exhibited pharmacological activities comparable to those of the prototype compound Pts in reducing cerebral infarction and alleviating neurological deficits. The tendency of improving CBF may be associated with their antioxidant and anti-inflammatory activities. Notably, compound NO. **3** showed markedly stronger activity, with more pronounced antioxidant and anti-inflammatory effects. Consistent with the results of the virtual screening and in vitro studies, compound NO. **3** emerged as the most promising candidate for further development.

### 2.5. Compound NO. ***3*** Improved Pathological Damage in the Hippocampus and Cortex of MCAO/R Rats

**Figure 15:**
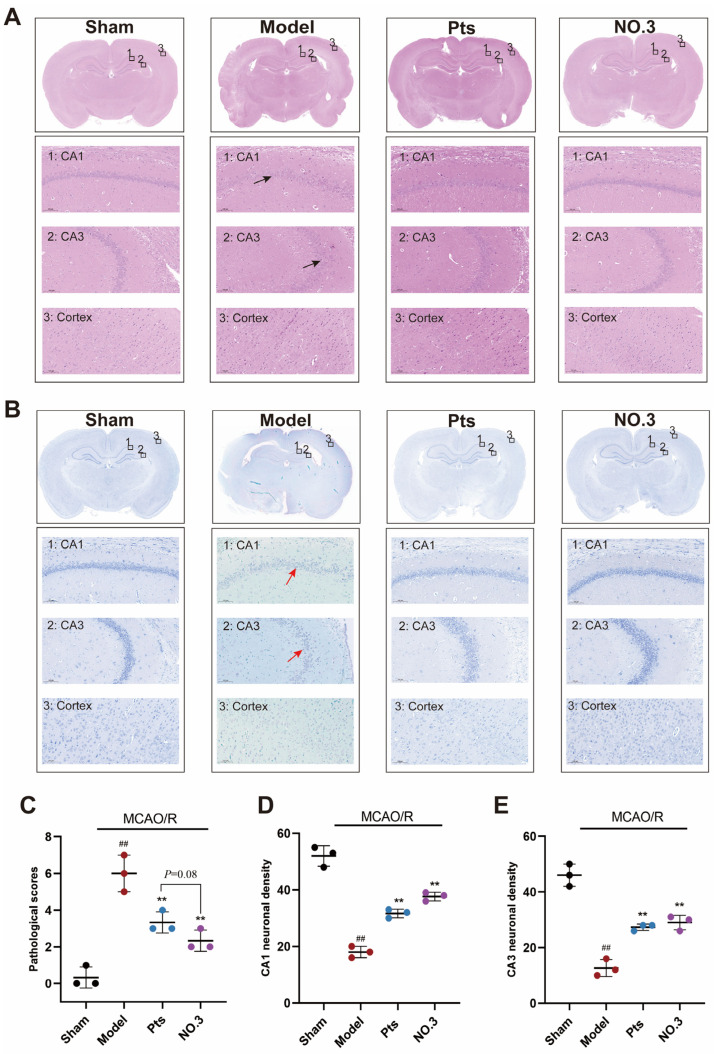
The histopathological protective effect of Pts and NO. **3** on MCAO/R rats. (**A**) Representative images of HE staining in the hippocampal CA1, CA3 and cortex (20× magnification, Scale Bar = 100 μm). (**B**) Representative images of Nissl staining in the hippocampal CA1, CA3 and cortex (20× magnification, Scale Bar = 100 μm). (**C**) Semi-quantitative scoring of HE staining. (**D**,**E**) Neuronal density in the hippocampal CA1 and CA3. Data are presented as the Mean ± SD of each group (n = 3). The black arrows indicate eosinophilic neuron, and the red arrows indicate Nissl body loss in the ischemic penumbra. ^##^ *p* < 0.01 vs. control group, ** *p* < 0.01 vs. model group.

Histopathological changes in the hippocampus and cortex were evaluated by HE and Nissl staining. MCAO/R injury induced pathological alterations, including an increased number of eosinophilic neurons, proliferation of activated astrocytes, and condensed, hyperchromatic nuclei (Figure 15A). Compared with the model group, NO. **3** (5 mg/kg) attenuated histopathological damage, as evidenced by improved cellular organization and reduced vacuolization. Semi-quantitative scoring based on the severity of pathological injury (Figure 15C) further demonstrated that NO. **3** exerted the most prominent protective effect on brain tissue (61.17% reduction of the model group).

The results of Nissl staining were presented in Figure 15B,D,E. Compared with the sham group, the model group exhibited a marked reduction in the number of Nissl bodies in the hippocampus, accompanied by severe neuronal damage and loosely arranged neurons in the cortex. Treatment with NO. **3** significantly attenuated neuronal loss in both the hippocampal CA1 and CA3 regions, and it preserved the neuronal morphology of the cortex. Moreover, the results of neuronal density showed that, compared to the model group, the Pts group and NO. **3** group increased by 75.93% and 109.26%, respectively, in the hippocampal CA1 and increased by 115.76% and 128.94%, respectively, in the hippocampal CA3. The results indicate that, NO. **3** could improve the integrity of the brain tissue structure, reduce the degree of brain edema, and enhance the survival status of neurons.

### 2.6. Acute Toxicity Assessment of Compound NO. ***3***

**Figure 16:**
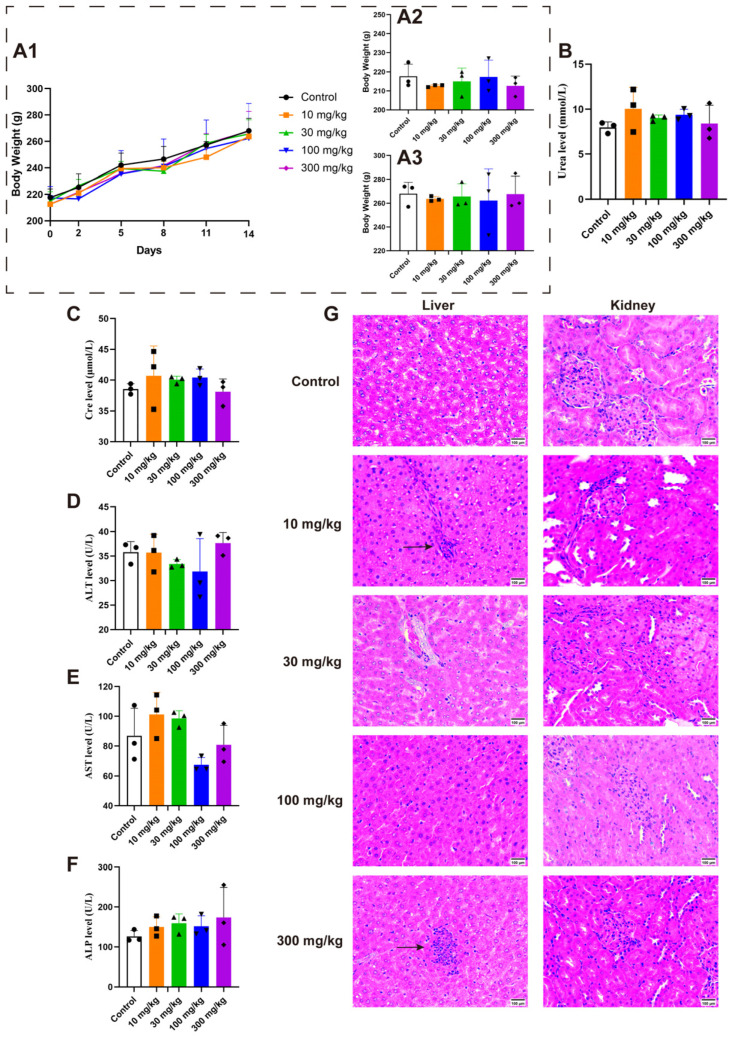
The acute intraperitoneal toxicity test of NO. **3** and dose of 10 mg/kg, 30 mg/kg, 100 mg/kg, and 300 mg/kg in female SD rats. (**A1**–**A3**) Body weight of different groups for 14 days (**A1**), at 0 day (**A2**), and at 14 days (**A3**). (**B**,**C**) Biochemical analysis of serum urea, Cre level in each group. (**D**–**F**) biochemical analysis of serum ALT, AST, ALP in each group. (**G**) HE staining images of liver and kidney in each group (×200, Scale Bar = 100 µm). The black arrows indicate inflammatory infiltrating cells. Data are presented as the Mean ± SD of each group (n = 3). No significant difference was observed among control group and drug administration groups. Cre, creatinine; ALT, alanine transaminase; AST, aspartate transaminase; ALP, alka-line phosphatase.

The preliminary safety of compound NO. **3** was further evaluated through an acute toxicity study using a stepwise procedure in accordance with OECD 423. After a single administration of 10, 30, 100, and 300 mg/kg, no obvious behavioral toxicity signs were observed during the 14-day observation period (Table S4). The body weight of all groups of rats increased, and there was no statistical difference between the treatment groups and the control group (Figure 16A1–A3). The serum biochemical analysis showed that the renal function indicators (Figure 16B,C) and hepatic function indicators (Figure 16D–F) of the drug administration group rats remained within normal physiological ranges, and there was no statistical difference compared with the control group (*p* > 0.05). In the histological analysis (Figure 16G), the control rats exhibited normal glomeruli and regularly arranged renal tubules in the kidney, clear nuclei, and an intact hepatic lobular structure in the liver. Similar features were observed in most treated rats, although mild pathological alterations were observed, such as inflammatory cell infiltration and renal tubular epithelial cell vacuolation.

## 3. Discussion

CIRI is characterized not only by neuronal death but also by profound microvascular dysfunction [26]. Increasing evidence indicates that oxidative stress, inflammatory response [27], and microcirculatory impairment [28] play a vital role in the progress of CIRI. Therefore, pharmacological strategies aimed at BMECs may provide novel therapeutic benefits [29].

### 3.1. Structural Optimization

As a dimethyl analog of resveratrol, Pts exhibits markedly enhanced lipophilicity attributable to its two methoxy substituents. Its low molecular weight and favorable BBB permeability have collectively caused extensive research attention [9]. Based on this scaffold, structural modification of Pts represents a rational strategy for the development of novel bioactive molecules.

A study showed that Pts at a dose of 3000 mg/kg for 28 days in Swiss mice did not cause significant changes in body weight, hematological parameters, and histopathology of the heart, liver, spleen, and kidney [30]. In addition, a clinical trial indicated that Pts is generally safe for use in humans up to 250 mg/day [31]. Pts demonstrates high safety and biological activity, which are important reasons for its consideration as an attractive lead structure for further structural optimization.

In recent years, a considerable number of studies have focused on the structural optimization of Pts. Studies have shown that the implantation of nitrogen-containing heterocyclic (piperidine, morpholine) on the side chain could extremely enhance the anti-tumor activity of Pts [32], suggesting that it is a general strategy to optimize the pharmacological properties of stilbenes. The Structural–Activity Relationship (SAR) analysis of this study illustrated the same principles. Compared with the prototype compound Pts, when R_3_ was the nitrogen-containing fragment (Figure 1), such as the 4-acetylmorpholine fragment in compound NO. **3** and the N-isopropylacetamide fragment in NO. **7**, NO. **3** and NO. **7** showed further increases in TI. Compound NO. **3**, bearing a morpholine amide substituent, exhibited the most pronounced activity. Morpholine, as a commonly used pharmacophore, possesses a favorable lipophilicity–hydrophilicity balance, an appropriate pKa, and a relatively stable chair conformation [33]. It has been reported to exhibit a wide range of biological activities, including anticancer, anti-inflammatory, antiviral, antioxidant, and antibacterial effects [34]. This modification may improve the lipophilicity–hydrophilicity balance of the molecule, enhance aqueous solubility and membrane permeability [34], and thereby contribute to improved BBB permeability and in vivo exposure [35]. Compound NO. **7** bearing an isopropyl amide substituent did not show enhanced activity compared with Pts, possibly due to increased steric hindrance [36]. Interestingly, when the two methoxy groups of NO. **1** on the aromatic ring (R_1_) were replaced by hydroxyl groups to produce NO. **5**, the in vivo efficacy was reduced. This observation highlights the importance of the methoxy substituents in maintaining pharmacological activity. Methoxy groups generally increase molecular lipophilicity and improve metabolic stability [37]. The increase in lipophilicity is beneficial for BBB permeability and enhances the effective exposure of the brain [38]. Notably, this feature is also considered one of the key reasons why Pts exhibits superior pharmacokinetic properties and biological activity compared with resveratrol [9]. Overall, these results suggest that maintaining the methoxy substituents on the aromatic ring and introducing nitrogen-atom-containing groups at R_3_ may represent an effective strategy for optimizing the biological activity of Pts [39].

### 3.2. Biological Activities Interpretation Based on ADMET Prediction

ADMET prediction results showed that the predicted probability of the NO. **1**, NO. **3**, NO. **5**, and NO. **7** compounds being P-gp substrates was low (<10%), suggesting that they are less likely to be P-gp-mediated efflux. This property may support their accumulation in the brain and thereby promote their cytoprotective activity [40], which was also reflected by the reduced LDH release. Compounds NO. **1**, NO. **3**, and NO. **7** exhibited high Caco-2 cell permeability, suggesting favorable intestinal absorption potential. In contrast, NO. **5** showed a lower predicted value (−5.293), which may partially affect its in vivo efficacy.

Adequate BBB permeability is generally regarded as an important prerequisite for CNS drug development, since the BBB represents the primary obstacle limiting drug delivery to the brain [41]. In terms of distribution, BOILED-EGG analysis indicated that NO. **1**, NO. **3**, and NO. **7** had good BBB permeability, as they were in the yolk zone in the plot. In BBB penetration prediction, NO. **3** had a much higher probability (~90%) of exhibiting a brain-to-blood ratio greater than 10%. NO. **5** showed the lowest predicted BBB penetration (probability of Log BB > −1 = 0.028), which may partially explain why it exhibited significant activity in vitro but failed to demonstrate clear anti-cerebral ischemic efficacy in vivo. NO. **1** and NO. **7** showed probabilities of only ~10%. These results suggest that NO. **3** may be more likely to enter the brain tissue, which may partly explain its stronger anti-cerebral ischemic activity observed in the in vivo experiments. Although NO. **1** and NO. **7** were predicted to possess relatively low BBB permeability, they still exhibited appreciable protective effects in vivo. This may be attributed to several possible factors. (1) ADMET prediction models provide only approximate estimations and may not fully reflect in vivo pharmacokinetics under pathological conditions [42]. (2) BBB integrity was disrupted, and the permeability increased during CIRI, which may facilitate drug entry into the brain [43]. (3) Ischemic injury induces dynamic alterations in the expression and functional activity of P-gp, which may further modulate drug entry and accumulation in brain tissue [44].

Regarding metabolism, NO. **3** exhibited relatively low predicted clearance and a longer half-life, suggesting favorable metabolic stability and prolonged systemic exposure. These pharmacokinetic characteristics may facilitate sustained drug levels in vivo and may partially contribute to the superior anti-cerebral ischemic activity [45] observed for NO. **3**.

### 3.3. Comprehensive Analysis Between In Vitro and In Vivo Pharmacodynamic Characteristics

Insufficient cerebral microcirculation perfusion is the initiating factor of CIRI [46,47]. Along with multiple pathological mechanisms such as dysfunction of BMECs, oxidative stress, and intense inflammatory response, which ultimately lead to neurological dysfunction and brain tissue necrosis [48]. The endothelial migration and angiogenesis are essential processes for post-ischemic vascular remodeling and microcirculatory recovery [49]. NO. **3** demonstrated consistent and superior pharmacological activities compared to Pts in both in vitro and in vivo. In vitro, NO. **1** and NO. **7** exhibited protective effects against OGD/R-induced hBMECs and promoted cell migration and tube formation, showing activities generally equivalent to those of Pts. In vivo, although NO. **1** and NO. **7** showed a modest trend toward CBF improvement, they still produced comparable cerebrovascular protective effects to those of Pts on infarct volume reduction, neurological function recovery, and the regulation of oxidative stress and inflammatory markers. The relatively weaker in vivo efficacy observed for NO. **1** and NO. **7** may be partly attributed to their limited BBB permeability, which could result in lower effective concentrations in the brain compared with NO. **3**. This possibility is supported by the ADMET prediction results, which suggested a lower BBB permeability for NO. **1** and NO. **7**. The results demonstrated that the structural modifications retained the key pharmacophoric features responsible for biological activity, allowing the derivatives to display similar activities [50]. The parent scaffold exerts cerebrovascular protection in vivo, while further optimization in the molecular framework is still required.

Interestingly, NO. **5** significantly restored endothelial migratory ability and angiogenic behavior in vitro, but did not demonstrate significant anti-ischemic efficacy in vivo, as evidenced by the lack of improvement in cerebral microcirculation and infarct volume. One possible explanation may be inferred from the ADMET prediction results. The relatively low lipophilicity of NO. **5** (NO. **5**: Log P = 3.183; Pts: Log P = 3.412) may limit its ability to penetrate the BBB. However, NO. **5** increased the survival rate in MCAO/R rats and reduced serum LDH and IL-10 levels significantly. LDH release reflects generalized cellular damage rather than exclusively neuronal necrosis [51]. Its decline may imply attenuation of secondary systemic stress responses induced by cerebral ischemia [52]. The normalization of IL-10 levels may indicate mitigation of excessive inflammatory signaling rather than direct immunosuppression [53,54]. These results suggested that NO. **5** may exert systemic cytoprotective and anti-inflammatory effects without directly ameliorating local ischemic injury. This discrepancy may be attributed to the complex physiological factors in vivo, including limited brain exposure, rapid metabolism, or insufficient tissue penetration [55].

### 3.4. Preliminary Safety Evaluation of NO. ***3***

Systemic administration of anti-ischemic agents requires BBB permeation for effective brain delivery [56,57], yet this constraint demands high circulating concentrations that compromise the safety profile. The narrow therapeutic window represents a dominant factor underlying the failure of anti-ischemic stroke drugs [58]. In addition to strong biological activities, preferable safety characteristics are equally important for potential therapeutic compounds [59].

In the limit test of NO. **3**, no mortality or overt signs of toxicity were observed at 300 mg/kg, indicating the LD_50_ value exceeded this dose level. ADMET prediction indicated a moderate risk of hepatotoxicity (H-HT = 0.663, DILI = 0.576), which is consistent with the observation that mild alterations were more frequently detected in the liver than in the kidney. Similar phenomena are frequently reported in acute toxicity studies and are generally regarded as having no toxicological significance [60].

The effect of oral administration is affected by bioavailability and liver first-pass effect, and the intraperitoneal injection route is usually selected to determine the intrinsic toxicity of chemicals. A study on the effect of different routes of administration (gavage, intramuscular, and intraperitoneal routes) on the LD_50_ of the ethanol extract of *Salvia przewalskii* Maxim was conducted. It was found that the LD_50_ of intraperitoneal injection was more than three times lower than that of oral administration, indicating that intraperitoneal injection had higher in vivo exposure and more obvious toxicity under the same conditions [61]. Therefore, it can be inferred that the LD_50_ of oral administration of NO. **3** will be much lower. The predicted rat oral acute toxicity (ROAT) value (0.118) further supports the relatively low acute toxicity of NO. **3**. Using the Globally Harmonized System (GHS) classification criteria for acute oral toxicity as a general reference framework, compound NO. **3** may be tentatively classified within Category 4 (300–2000 mg/kg) or a lower hazard class [62]. Furthermore, with an estimated therapeutic index exceeding 60 times calculated from the effective dose (5 mg/kg) relative to the limit dose (more than 300 mg/kg), NO. **3** demonstrates a relatively wide preliminary safety margin, supporting its potential for further pharmaceutical development. These observations imply that structural modification of the prototype drug Pts did not introduce obvious safety liabilities. This favorable safety profile is particularly encouraging for further development.

## 4. Materials and Methods

### 4.1. Preparation of Pts and Its Derivatives

Pterostilbene (purity > 98%, HPLC grade) were purchased from Shanghai Bide Pharmaceutical Technology Co., Ltd. (Shanghai, China). Representative active compounds (NO. **1**, NO. **3**, NO. **5**, and NO. **7**) were fully characterized by ^1^H-NMR.

### 4.2. Biological Assay

#### 4.2.1. ADMET Studies and BOILED-EGG Plot

The SMILES numbers of derivatives were input into the ADMETlab 2.0 website (https://admetmesh.scbdd.com/, accessed on 9 July 2025) to obtain the parameters of absorption, distribution, metabolism, excretion, and toxicity characteristics of Pts and its derivatives. The SMILES numbers were also input into the SwissADME website (https://www.swissadme.ch/, accessed on 21 July 2025) to obtain the predicted results of blood–brain barrier permeability and gastrointestinal absorption of each derivative.

#### 4.2.2. Cell Culture

hBMECs and SH-SY5Y cells were obtained from the Cell Center of Peking Union Medical College. Both cells were cultured in DMEM: F12 medium (Biological Industries, Cromwell, CT, USA) supplemented with 10% FBS (PAN-Biotech, Aidenbach, Germany), 100 U/mL penicillin and 0.1 mg/mL streptomycin (Solarbio, Beijing, China) and incubated at 37 °C in a humidified atmosphere containing 5% CO_2_.

#### 4.2.3. Establishment of OGD/R Model

The OGD/R model was constructed based on the previous methods [18]. Briefly, the culture medium was replaced with Earle’s balanced salt solution, and the cells were placed in an anaerobic incubator (COY Laboratory, Grass Lake, MI, USA) for 2 h. Then, the cells were removed, divided into the model group (FBS-free medium) and drug administration group (drug added in FBS-free medium), and further cultured under normal conditions for a certain time.

#### 4.2.4. MTT Assay

Cells were seeded into 96-well plates and cultured. Initially, 10 μL of MTT solution (Solarbio, Beijing, China) was added to each well and incubated at 37 °C for 4 h in the dark. The culture medium was then removed, and 100 μL of DMSO (Macklin, Shanghai, China) was added to dissolve the formazan crystals and then vibrated for 5 min in the dark. The cell viability was quantified by measuring absorbance at 490 nm using a microplate reader (Tecan, Männedorf, Switzerland).

#### 4.2.5. Maximal Recovery Rate and Therapeutic Index

Maximal recovery rate = (maximal cell viability of treatment group − cell viability of model group)/(100% − cell viability of model group) × 100%.

Based on the effects of derivatives on the cell viability of normal or after OGD/R cells, the IC_50_ and EC_50_ were evaluated. TI = IC_50_/EC_50_ × 100%.

#### 4.2.6. LDH Release Assay

The hBMECs supernatant was collected and then centrifuged at 3000 rpm after OGD/R and incubated with drugs for 24 h. The rat serum was obtained after continuous administration for 7 days. The LDH leakage of each group was determined according to the instructions of the LDH kit (Nanjing Jiancheng Bioengineering Institute, Nanjing, China).

#### 4.2.7. Hoechst33324/PI Assay

After hBMECs were treated with OGD/R and drug administration incubation, they were gently rinsed with PBS. Then, Hoechst33342and Propidium Iodide (PI) were added sequentially to incubate at 4 °C for 15 min. hBMECs were washed twice with PBS and observed under a fluorescence microscope (Nikon, Tokyo, Japan).

#### 4.2.8. Scratch and Transwell Migration Assay

Cells were cultured in a 6-well plate and cultured to 95% confluence. After OGD, a 200 µL sterile pipette was used to draw a vertical line in each well. The cell fragments were washed under the PBS solution gently and then incubated in the corresponding culture medium for 24 h. The scratch area was photographed at the detection points. The ImageJ software (v1.53e) was used to measure the scratch area. The area of migration = (scratch area at 0 h−scratch area at 24 h)/(scratch area at 0 h) × 100%.

For the Transwell assay, hBMECs were seeded in the Transwell upper chambers, and 600 μL of 10%FBS-DMEM was added into the lower chambers. After 6 h, the migrated cells were stained by 1% crystal violet. The number of cells migrating to the lower chamber was calculated using Image J software.

#### 4.2.9. Tube Formation Assay

HBMECs were seeded onto Matrigel-coated (BioCoat, Horsham, PA, USA) 96-well plates and incubated for 6 h. The formation of tubes was observed under an inverted microscope (Olympus, Tokyo, Japan) and photographed for statistical analysis of the number of nodes, branches, and total branching length.

#### 4.2.10. Oxidative Stress Indicators Detection

The supernatants of OGD/R cells and the rat serum were collected as total cellular protein. The levels of oxidative stress indicators were subsequently determined according to the instructions of the CAT kit (Nanjing Jiancheng Bioengineering institute, Nanjing, China), SOD kit (Solarbio, Beijing, China), MDA kit (Yuanye Bio-Technology, Shanghai, China).

#### 4.2.11. Experimental Animals

Male Sprague-Dawley rats (280–300 g) were purchased from Huafukang Co., Ltd. (Beijing, China; certificate NO. SCXK2024-003). The animal care and experiments were all followed by the Ethics Committees of the Institute of Medicinal Plant Development, Chinese Academy of Medical Sciences, and Peking Union Medical College.

#### 4.2.12. MCAO/R Surgery

The MCAO/R model was established in SD rats according to a previous study [63]. After adaptive feeding, the rats at same age and of similar weight were fasted for 12 h before the MCAO/R operation. The rats were anesthetized with Zoletil 50 (ip., 40 mg/kg) and then fixed in a supine position. The right common carotid artery (CCA), internal carotid artery (ICA), and external carotid artery (ECA) were exposed and separated. The proximal segments of the CCA and the ECA were ligated. A 0.38 mm silicone-coated monofilament was introduced through the ICA and advanced to the proximal origin of the middle cerebral artery (MCA). During this period, the body temperature of the rats was maintained at a normal level. After 1.5 h, the monofilament was slowly withdrawn to restore blood flow. The sham group only performed vascular separation without inserting the monofilament.

#### 4.2.13. Experimental Groups and Drug Administration

The positive drug Pts (purity > 98% by HPLC) was purchased from Shanghai Bide Pharmaceutical Technology Co., Ltd. (Shanghai, China). The rats were at the same age and similar weight after adaptive feeding. At the beginning of the operation, some rats were selected for the sham operation group. After MCAO/R surgery, the modified neurological severity score (mNSS) was conducted when the rat regained consciousness. Based on the scores and the results of cerebral blood flow (CBF), the model’s success was preliminarily judged. After excluding the rats with unsuccessful modeling, the remaining rats were randomly divided into the model group, Pts + MCAO/R group, NO. **1** + MCAO/R group, NO. **3** + MCAO/R group, NO. **5** + MCAO/R group, and NO. **7** + MCAO/R group. The drugs were administered within 6 h of reperfusion, and the administration method for each group of rats was intraperitoneal injection, with a dose of 5 mg/kg for all groups. The rats in the sham group, model group, and drug administration groups received intraperitoneal injection of the same volume of solvent (5% PEG400-95% physiological saline) at the same time. These medications were subsequently administered daily for 7 days.

#### 4.2.14. Modified Neurological Severity Score (mNSS) Test

The degree of neurological injury was evaluated using the mNSS for subsequent experiments. Rats with a neurological function score of 7–15 were selected for the subsequent research. On days 1, 3, and 7 after the operation, the mNSS were assessed for MCAO/R rats to observe the effect of the drug administration on the recovery of the rats’ neurological function.

#### 4.2.15. Laser Speckle Imaging Technique for Measuring CBF

The laser speckle blood flow imaging instrument (PeriCam PSI System, Perimed, Järfälla, Sweden) was used to measure the cerebral blood flow (CBF) of rats at these points: before the operation (normal), after the insertion of the monofilament (ischemia), after the removal of the line plug (reperfusion), and after drug administration (7 days).

#### 4.2.16. 2,3,5-Triphenyltetrazolium Chloride (TTC) Staining

The brain was sliced into five coronal sections (approximately 2 mm thick) in the direction of the forehead–occipital region, and incubated in 1% TTC at 37 °C. The cerebral infarct volume was quantified by ImageJ software.

#### 4.2.17. Enzyme-Linked Immunosorbent Assay (ELISA)

Rat serum levels of IL-1β, IL-6, TNF-α, and IL-10 were measured using assay kit IL-1β (ml037361, Shanghai Enzyme-linked Biotechnology Co., Ltd., Shanghai, China), IL-6 (ml102828, Shanghai Enzyme-linked Biotechnology Co., Ltd.), TNF-α (ml002859, Shanghai Enzyme-linked Biotechnology Co., Ltd.), and IL-10 (ml037371, Shanghai Enzyme-linked Biotechnology Co., Ltd.), respectively, according to the manufacturer’s instructions.

#### 4.2.18. HE/Nissl Staining

Fresh brain tissues were fixed in 4% paraformaldehyde at 4 °C. Then, they were dehydrated through a graded ethanol series, cleared in xylene, and embedded in paraffin. Coronal sections were prepared using a microtome. For hematoxylin-eosin (HE) staining, paraffin sections were deparaffinized, rehydrated, and stained with hematoxylin followed by eosin. Nissl staining was performed using cresyl violet solution. For HE analysis, the degree of pathological injury was evaluated using a semi-quantitative scoring system. Nissl staining was performed to assess neuronal survival in hippocampal subregions.

#### 4.2.19. Acute Intraperitoneal Toxicity of Compound NO. **3**

Acute toxicity tests were carried out followed by the guidelines of the Organization for Economic Co-Operation and Development (OECD) guidelines 423 [64] and *Guidance on Dose Level Selection for Regulatory General Toxicology Studies for Pharmaceuticals* [65], with slight modifications. Female Sprague-Dawley rats (8 weeks) were purchased from Beijing Huafukang Co., Ltd. (Beijing, China; certificate NO. SCXK2024-003). The rats were randomly divided into five groups (3 rats per group): the normal group, 10 mg/kg administration group, 30 mg/kg, 100 mg/kg, and 300 mg/kg administration group. The acute toxicity assessment was performed using a dose-escalation design. Rats were fasted overnight for 12 h with free access to water and intraperitoneally administrated at a single dose of 5% PEG400-95% physiological saline (control vehicle) or 10 mg/kg of NO. **3**. Rats were closely monitored during the first 4 h for clinical signs of toxicity, including changes in activity, alertness/sleeping, breathing, eating, and any abnormal behaviors. Animals were further observed for up to 24 h to detect delayed toxic responses or mortality. If no apparent toxic effects were observed within the 24 h observation period, a separate group of rats was administered a higher dose on the following day using the same procedure. The rats in the experimental group were administered once and were observed for 14 days. At the end of the experiment, the rats were anesthetized and executed. The blood was collected the biochemical parameters such as urea, creatinine (Cre), alanine transaminase (ALT), aspartate transaminase (AST), and alkaline phosphatase (ALP) were investigated by using a clinical chemistry system AU 480 (Beckman Coulter, Tokyo, Japan). The liver and kidneys were processed for HE staining for histopathological examination.

### 4.3. Statistical Analysis

Data and graphs were statistically analyzed using GraphPad Prism (version 8.4.2). The final results were presented as mean ± standard deviation (Mean ± SD). The IC_50_ values and EC_50_ values of each compound were fitted in GraphPad Prism. The differences between data from two groups were analyzed using the *t*-test; the differences between data from multiple groups were analyzed using one-way ANOVA and Tukey’s post-hoc test. In all analyses, *p* < 0.05 was considered significant.

## 5. Conclusions

In this study, Pts was utilized as the chemical scaffold to design and screen a series of derivatives. The anti-cerebral ischemic activities of these compounds were systematically evaluated through virtual screening, in vitro, and in vivo experiments. ADMET prediction indicated that NO. **3** possessed favorable gastrointestinal absorption and BBB permeability. In vitro experiments demonstrated that NO. **1**, NO. **3**, NO. **5**, and NO. **7** exhibited higher TI than the parent compound Pts. These compounds significantly reduced LDH release; promoted the horizontal and vertical migration of BMECs; enhanced tube formation ability; increased the levels of the antioxidant enzymes SOD and CAT; and decreased the lipid peroxidation product MDA in OGD/R-injured hBMECs. In MCAO/R rats, compound NO. **3** significantly increased CBF, reduced cerebral infarct volume, improved neurological function, decreased LDH release, and regulated oxidative stress and inflammatory factors. Compounds NO. **1** and NO. **7** displayed pharmacological activities comparable to those of Pts, whereas NO. **3** exhibited superior efficacy. NO. **3** has a high safety profile, and its LD_50_ was determined to be >300 mg/kg. These findings suggest that NO. **3** may ameliorate cerebral microcirculation and neurological function by exhibiting potent antioxidant and anti-inflammatory activities. The protective effects of NO. **3** on BMECs may contribute to the maintenance of NVU function and enhance the integrity of BBB. In conclusion, these results implied the potential of NO. **3** as a promising anti-ischemic drug candidate. Further dose–response studies and exploration of the underlying molecular mechanisms of NO. **3** will be conducted to more comprehensively assess its efficacy compared with Pts.

## Figures and Tables

**Table 1:** The fragments of pterostilbene (Pts) and its derivatives.

Compounds	R_1_	R_2_	R_3_	SMILES
Pts	CH_3_	H	H	COC1=CC(/C=C/C2=CC=C(O)C=C2)=CC(OC)=C1
NO. **1**	CH_3_	H	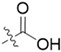	COC1=CC(/C=C/C2=CC=C(O)C(C(O)=O)=C2)=CC(OC)=C1
NO. **3**	CH_3_	H	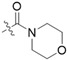	COC1=CC(/C=C/C2=CC=C(O)C(C(N3CCOCC3)=O)=C2)=CC(OC)=C1
NO. **5**	H	H	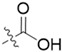	OC1=CC(/C=C/C2=CC=C(O)C(C(O)=O)=C2)=CC(O)=C1
NO. **7**	CH_3_	H	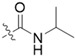	OC1=C(C(NC(C)C)=O)C=C(/C=C/C2=CC(OC)=CC(OC)=C2)C=C1

Pts, pterostilbene; SMILES, simplified molecular input line entry system.

**Table 2:** The maximal recovery rate and the concentration of derivatives in OGD/R induced SH-SY5Y cells or hBMECs.

Compounds	Maximal Recovery Rate of hBMECs (%)	Maximal Recovery Rate of SH-SY5Y (%)	More Sensitive Cells
NO. **1**	34.63 (15.0 μM)	24.64 (6.25 μM)	hBMECs
NO. **3**	53.44 (15.0 μM)	52.49 (6.25 μM)	insignificance
NO. **5**	64.64 (60.0 μM)	41.56 (12.5 μM)	hBMECs
NO. **7**	33.32 (7.5 μM)	29.75 (6.25 μM)	insignificance

hBMECs, human brain microvascular endothelial cells.

**Table 3:** Maximal recovery rate and TI of positive control Pts and its derivatives under the condition of OGD/R-induced hBMECs injury models.

Compounds	Cell Viability (%)		(Maximal Recovery Rate, %)/ (Concentration, μM)	IC_50_ (μM)	EC_50_ (μM)	TI
	Model	Drug				
Pts	60.95	93.04	53.17 (20 μM)	62.43	8.18	7.63
NO. **1**	48.73	63.56	28.94 (10 μM)	78.10	4.27	18.29
NO. **3**	53.05	72.66	41.78 (15 μM)	85.74	3.50	24.50
NO. **5**	50.60	82.53	64.64 (60 μM)	313.70	11.75	26.70
NO. **7**	47.29	65.56	34.67 (10 μM)	235.40	7.69	30.61

IC_50_, half inhibition concentration; EC_50_, half effective concentration; TI, therapeutic index.

## Data Availability

The original contributions presented in this study are included in the article. Further inquiries can be directed to the corresponding authors.

## Supplementary Materials

The following supporting information can be downloaded at: https://www.mdpi.com/article/10.3390/ijms27104512/s1.

## Abbreviations

The following abbreviations are used in this manuscript:

BBB	Blood–brain barrier
BMECs	Brain microvascular endothelial cells
Caco-2	Human colon adenocarcinoma cell lines
CAT	Catalase
CBF	Cerebral blood flow
CCA	Carotid artery
CIRI	Cerebral ischemia-reperfusion injury
CNS	Central nervous system
ECA	External carotid artery
GHS	Globally Harmonized System
ICA	Internal carotid artery
LDH	Lactate dehydrogenase
MCAO/R	Middle cerebral artery occlusion/reperfusion
MDA	Malondialdehyde
NVU	Neurovascular unit
OGD/R	Oxygen-glucose deprivation/reperfusion
P-gp	P-glycoprotein
Pts	Pterostilbene
ROAT	Rat oral acute toxicity
SAR	Structural–Activity Relationship
SMILES	Simplified Molecular Input Line-entry System
SOD	Superoxide dismutase
THF	Tetrahydrofuran
TI	Therapeutic index
TTC	2,3,5-triphenyltetrazolium chloride
