# Supplementary material for: Structural Optimization of Pterostilbene, a Promising Lead Molecule, and Evaluation of Its Derivatives via ADMET Prediction and In Vitro/In Vivo Anti-Cerebral Ischemic Activity
Source: Int J Mol Sci. 2026 May 18;27(10):4512. doi: 10.3390/ijms27104512 (PMC13207910; doi:10.3390/ijms27104512)
Supplement: Supplementary file 1 [file ijms-27-04512-s001.zip › ijms-4235854-supplementary.pdf]

Supplementary Materials

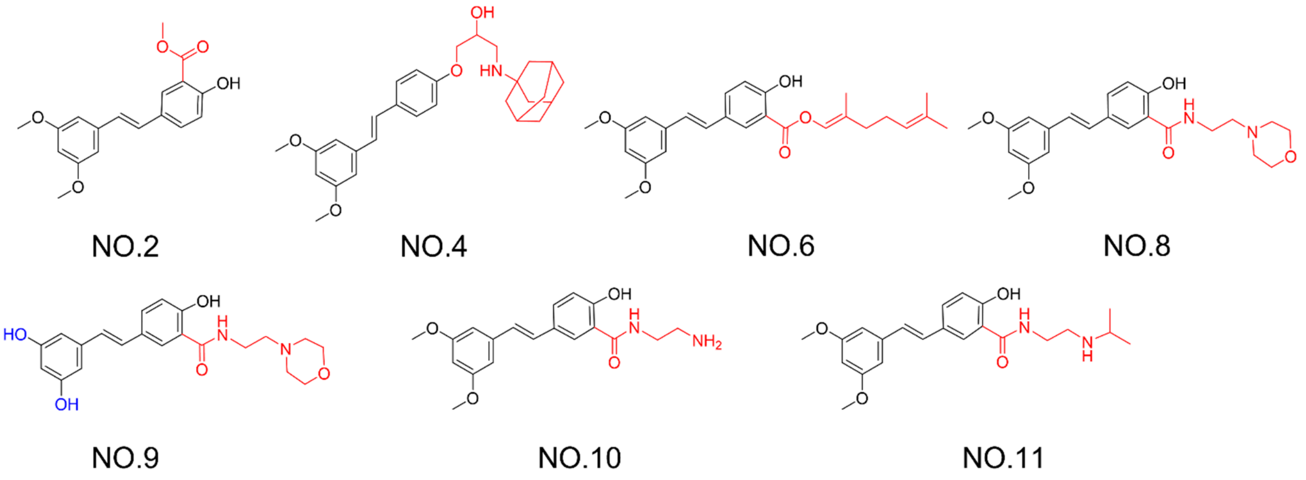

Figure S1. The structure of other Pts derivatives.

A: ^{1}H NMR Spectra for compound NO.1

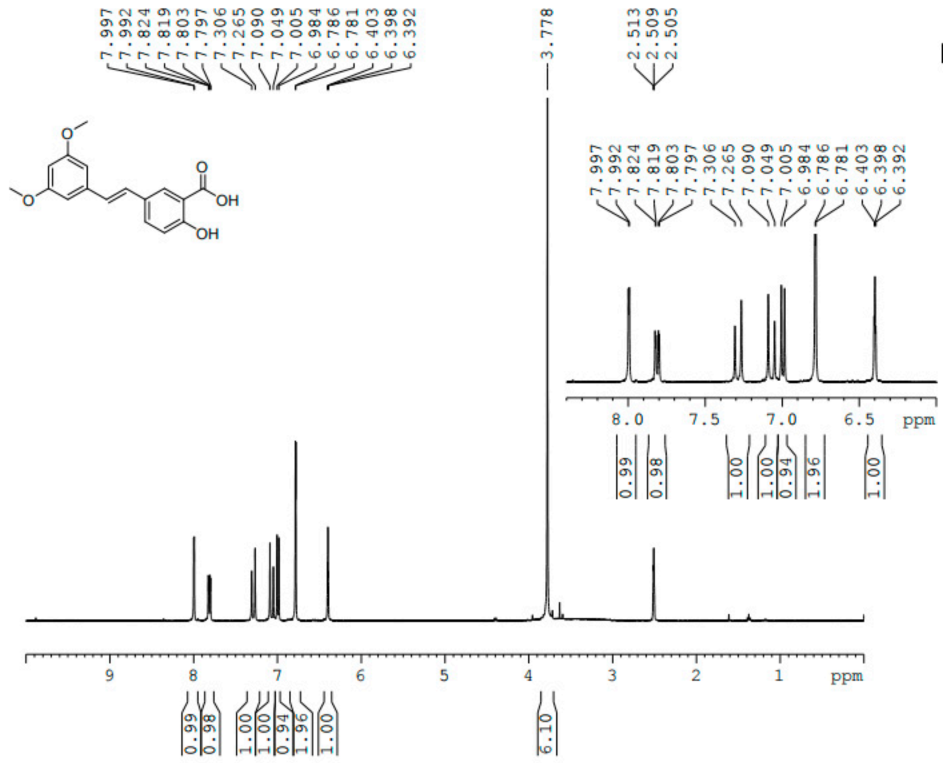

^{1}H NMR (400M, DMSO-d6) δ:7.99 (1H, d), 7.82-7.80 (1H, dd), 7.31-7.27 (1H, d), 7.09-7.05 (1H, d), 7.10-6.98 (1H, d), 6.78 (2H, d), 6.40-6.39 (1H, t), 3.78(6H, s).

B: ^{1}H NMR Spectra for compound NO.3

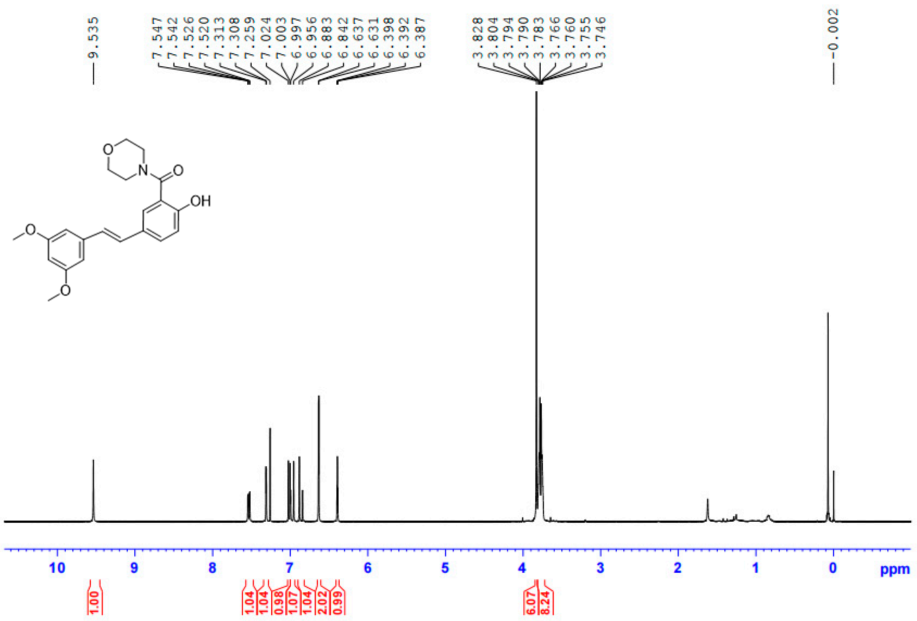

^{1}H NMR(400M, $CDCl_3$) δ:9.53 (1H, s), 7.55-7.52 (1H, dd)，7.31-7.30 (1H, d)，77.02-7.00 (1H, d)，6.96 (1H, s)，6.88-6.84 (1H, d)，6.64-6.63 (2H, d)，6.40-6.39 (1H, t)，3.83 (6H, s)，3.80-3.75 (8H, brs).

C: ^{1}H NMR Spectra for compound NO.5

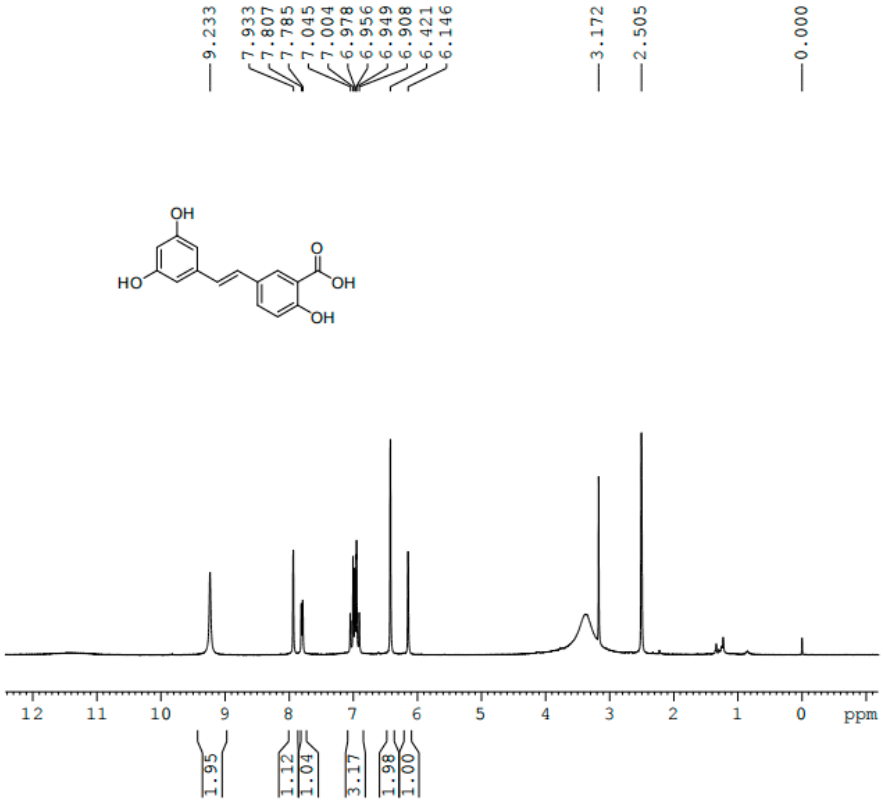

^{1}H-NMR (400M, DMSO-d6) δ:9.23 (1H, brs), 7.93 (1H, s), 7.81-7.79 (1H, d),7.05-6.91 (3H, m), 6.42 (2H, s), 6.15(1H, s).

D: ^{1}H NMR Spectra for compound NO.7

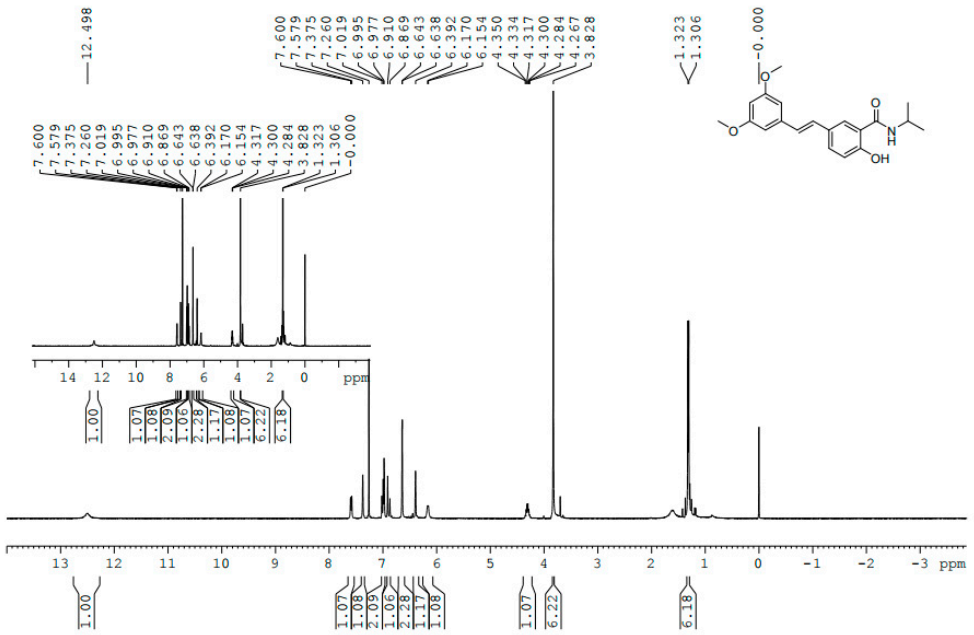

^{1}H-NMR (400M, $CDCl_3$) δ:12.49 (1H, br), 7.60-7.58 (1H, d), 7.38(1H, s), 7.02-6.98 (2H, t), 6.91-6.87 (1H, d), 6.64-6.38 (2H, d), 6.39 (1H, s), 6.17-6.15 (1H, d), 4.33-4.27(1H, m), 3.83 (6H, s), 1.32-1.31(6H, d).

Figure S2. ^{1}H-NMR Spectra for Pts derivatives (A) NO.1, (B) NO.3, (C) NO.5, and (D) NO.7.

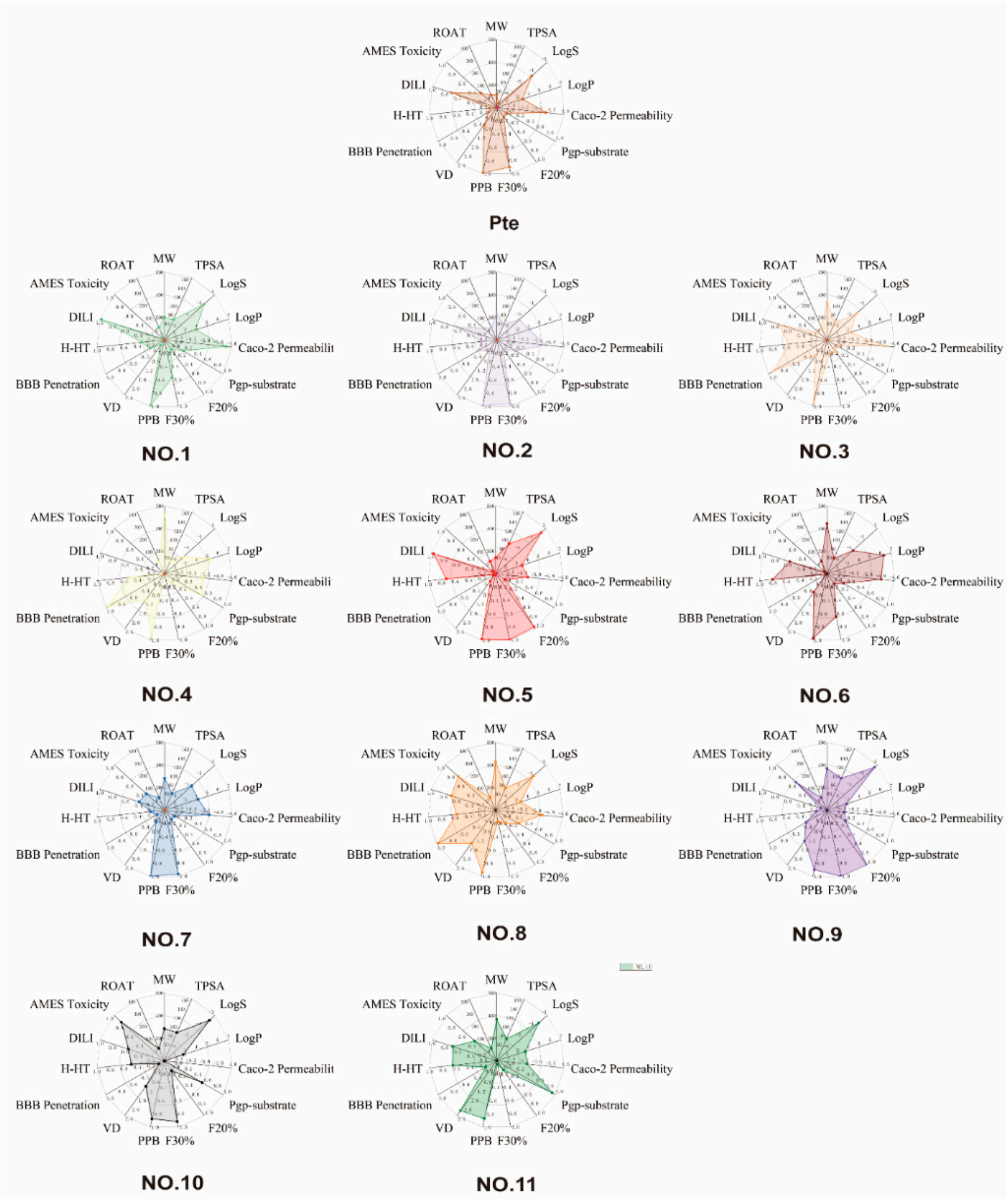

Figure S3. A radar plot displayed the key ADMET parameters of all derivatives predicted using ADMETlab 2.0.

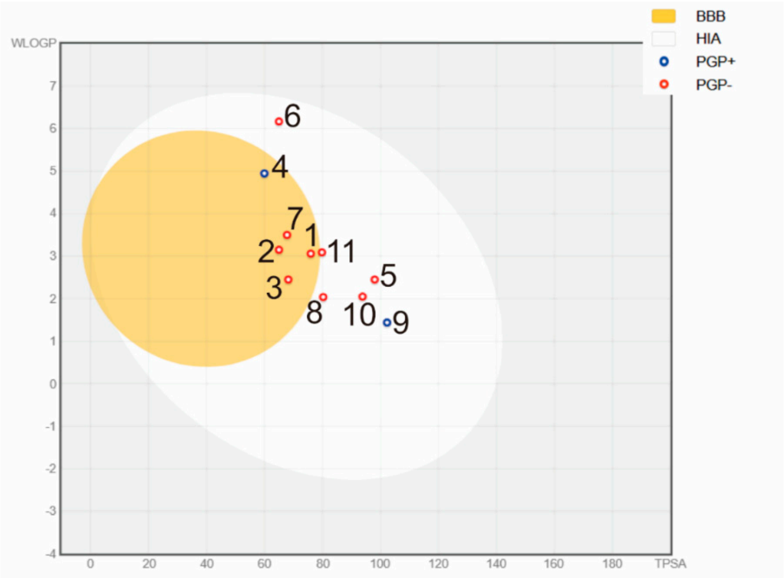

Figure S4. A BOILED-EGG model of Pts derivatives.

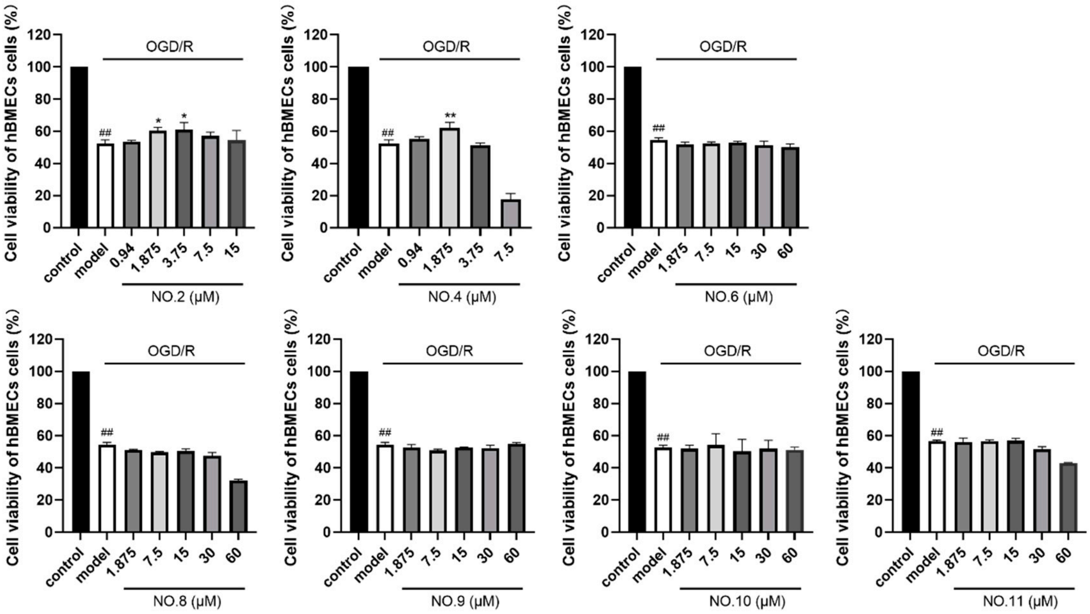

Figure S5. Preliminary screening of other derivatives in OGD/R-induced hBMECs injury models after 24 h co-incubation.

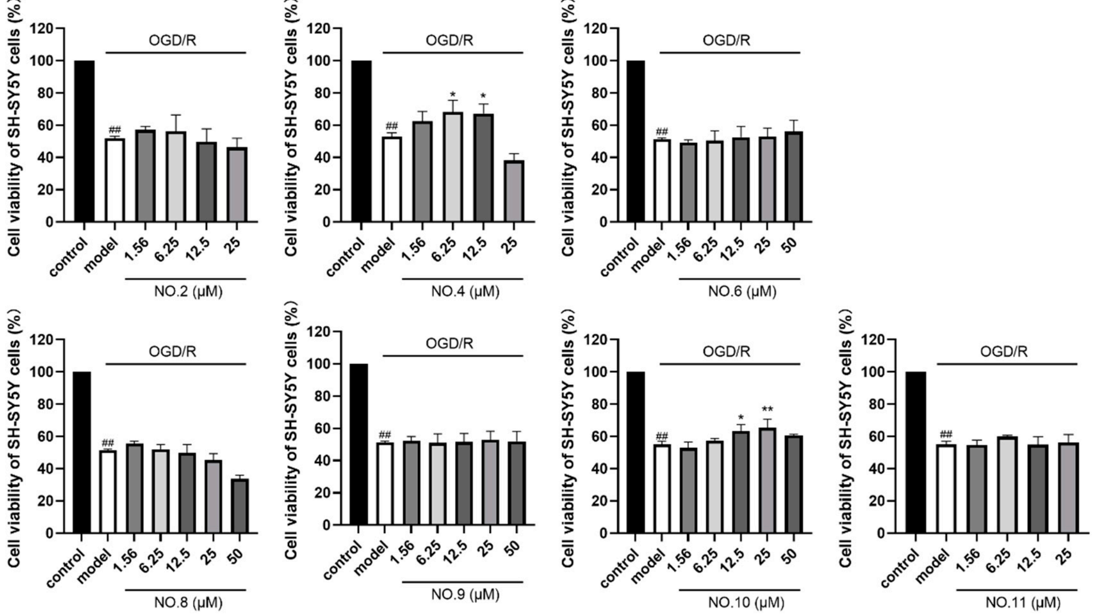

Figure S6. Preliminary screening of other derivatives in OGD/R-induced SH-SY5Y injury models after 24 h co-incubation.

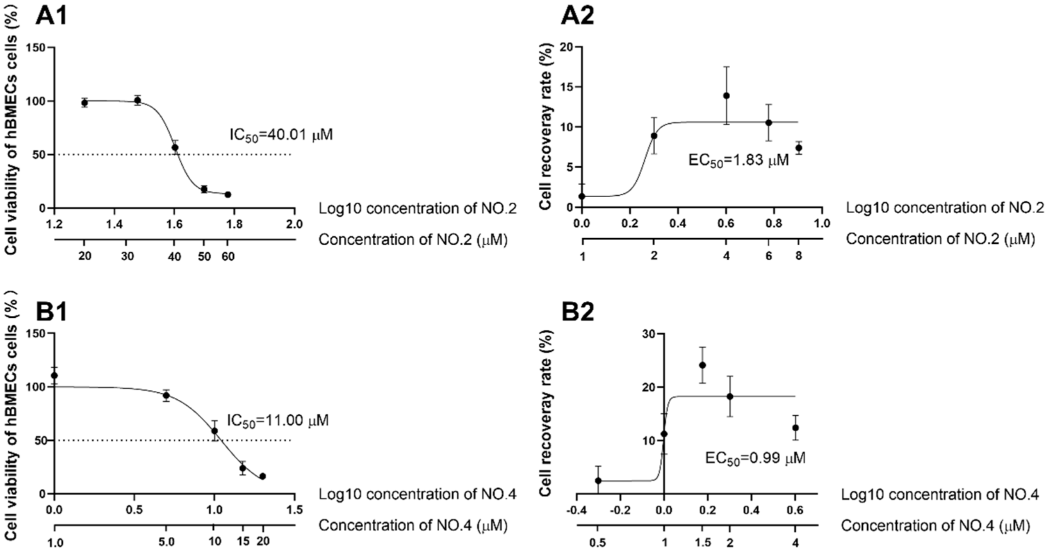

Figure S7. (A) The IC_{50} value and (B) the EC_{50} value of NO.2 and NO.4 effect on normal or OGD/R-induced hBMECs for 24 h.

Table S1 The ADMET prediction results of Pts and its derivatives.

	Index	Compound											
		NO.1	NO.2	NO.3	NO.4	NO.5	NO.6	NO.7	NO.8	NO.9	NO.10	NO.11	Pts
Physicochemical property	MW	300.10	314.12	369.16	463.27	272.07	422.21	341.16	412.20	384.17	342.16	384.20	256.11
	nHA	5	5	6	5	5	5	5	7	7	6	6	3
	nHD	2	1	1	2	4	1	2	2	4	4	3	1
	nROT	5	6	6	10	3	10	7	9	7	8	10	4
	TPSA	75.99	64.99	68.23	59.95	97.99	64.99	67.79	80.26	102.26	93.81	79.82	38.69
	LogS	-4.116	-5.207	-4.696	-5.524	-3.296	-5.194	-4.853	-4.117	-3.059	-3.415	-3.729	-4.236
	LogP	3.979	3.997	3.251	5.290	3.183	6.236	4.11	3.406	2.498	2.757	3.709	3.412
Absorption	Caco-2 Permeability	-4.785	-4.869	-4.863	-5.025	-5.293	-4.872	-4.939	-5.129	-5.511	-5.701	-5.316	-4.870
	Pgp-substrate	0.012	0.179	0.004	0.564	0.004	0.084	0.014	0.092	0.061	0.571	0.663	0.006
	F20%	0.003	0.004	0.151	0.003	0.973	0.021	0.002	0.313	1.000	0.017	0.002	0.010
	F30%	0.908	0.960	0.568	0.008	0.997	0.770	0.963	0.526	0.998	0.916	0.285	0.899
Distribution	PPB	98.90%	98.78%	95.31%	93.99%	95.98%	97.47%	100%	94.43%	76.17%	87.62%	87.69%	99.00%
	VD	0.236	0.446	0.686	1.148	0.312	0.798	0.517	1.447	1.355	1.113	2.183	0.776
	BBB Penetration	0.107	0.307	0.829	0.967	0.028	0.016	0.131	0.963	0.282	0.090	0.116	0.141
Excretion	CL	3.264	9.005	7.664	9.174	8.193	8.302	5.756	10.167	11.295	9.075	7.574	10.189
	$T_{1/2}$	0.841	0.580	0.795	0.044	0.928	0.244	0.274	0.488	0.740	0.663	0.314	0.764
Toxicity	H-HT	0.486	0.218	0.663	0.529	0.777	0.870	0.206	0.510	0.138	0.490	0.597	0.101
	DILI	0.970	0.616	0.576	0.033	0.958	0.447	0.398	0.378	0.044	0.555	0.462	0.700
	AMES	0.012	0.247	0.112	0.013	0.009	0.017	0.364	0.405	0.117	0.852	0.211	0.313
	ROAT	0.041	0.026	0.118	0.019	0.097	0.016	0.028	0.147	0.111	0.245	0.244	0.017

Table S2. The maximal recovery rate and the concentration of NO.2, NO.4, and NO.10 in OGD/R induced SH-SY5Y cells or hBMECs.

Compounds	Maximal recovery rate of hBMECs (%)	Maximal recovery rate of SH-SY5Y (%)	More sensitive cells
NO.2	14.76 (3.75 μM)	-	-
NO.4	16.77 (1.875 μM)	32.32 (6.25 μM)	SH-SY5Y
NO.10	-	23.22 (25.0 μM)	-

Table S3. Maximal recovery rate and TI of NO.2 and NO.4 under the condition of OGD/R-induced hBMECs injury models.

**Compounds**	**Cell viability (%)**		**(Maximal recovery rate, %) / (Concentration, μM)**	**IC_{50} (μM)**	**EC_{50} (μM)**	**TI**
	**Model**	**drug**				
NO.2	52.35	58.97	13.89 (3.75 μM)	40.01	1.83	21.91
NO.4	52.35	63.86	24.15 (1.5 μM)	11.00	0.99	11.11

Table S4. Clinical signs of toxicity in Group CN (control), 10 mg/kg, 30 mg/kg, 100 mg/kg, and 300 mg/kg during the acute intraperitoneal toxicity study of NO.3 following by the guideline OECD 423 (n=3). Normal clinical signs were recorded as N, abnormalities are recorded as A.

Signs	30 min					4 h					24 h					7 d					14 h				
	CN	10	30	100	300	CN	10	30	100	300	CN	10	30	100	300	CN	10	30	100	300	CN	10	30	100	300
Activity	N	N	N	N	N	N	N	N	N	N	N	N	N	N	N	N	N	N	N	N	N	N	N	N	N
Breathing	N	N	N	N	N	N	N	N	N	N	N	N	N	N	N	N	N	N	N	N	N	N	N	N	N
Movement/ trembling	N	N	N	N	N	N	N	N	N	N	N	N	N	N	N	N	N	N	N	N	N	N	N	N	N
Eating	N	N	N	N	N	N	N	N	N	N	N	N	N	N	N	N	N	N	N	N	N	N	N	N	N
Drinking	N	N	N	N	N	N	N	N	N	N	N	N	N	N	N	N	N	N	N	N	N	N	N	N	N
Alertness	N	N	N	N	N	N	N	N	N	N	N	N	N	N	N	N	N	N	N	N	N	N	N	N	N
Body weight	N	N	N	N	N	N	N	N	N	N	N	N	N	N	N	N	N	N	N	N	N	N	N	N	N
Skin	N	N	N	N	N	N	N	N	N	N	N	N	N	N	N	N	N	N	N	N	N	N	N	N	N
Eyes	N	N	N	N	N	N	N	N	N	N	N	N	N	N	N	N	N	N	N	N	N	N	N	N	N
Faeces	N	N	N	N	N	N	N	N	N	N	N	N	N	N	N	N	N	N	N	N	N	N	N	N	N

Animals：SD female rats (8 weeks), SPF degree.
